# The influence of precipitation timing and amount on soil microbial community in a temperate desert ecosystem

**DOI:** 10.3389/fmicb.2023.1249036

**Published:** 2023-09-07

**Authors:** Yao Xiao, Fang Bao, Xiaotian Xu, Ke Yu, Bo Wu, Ying Gao, Junzhong Zhang

**Affiliations:** ^1^Key Laboratory of Forest Disaster Warning and Control in Yunnan Province, Faculty of Biodiversity Conservation, Southwest Forestry University, Kunming, China; ^2^Institute of Desertification Studies, Chinese Academy of Forestry, Beijing, China; ^3^Institute of Ecological Conservation and Restoration, Chinese Academy of Forestry, Beijing, China; ^4^Beijing Academy of Forestry and Pomology Sciences, Beijing, China; ^5^School of Environment and Energy, Shenzhen Graduate School, Peking University, Shenzhen, China; ^6^Key Laboratory of Forest Resources Conservation and Utilization in the Southwest Mountains of China Ministry of Education, Southwest Forestry University, Kunming, China; ^7^Key Laboratory of State Forestry and Grassland Administration on Highly-Efficient Utilization of Forestry Biomass Resources in Southwest China, Southwest Forestry University, Kunming, China

**Keywords:** precipitation increase, precipitation time, temperate desert, bacterial and fungal communities, Illumina sequencing

## Abstract

**Introduction:**

Global climate change may lead to changes in precipitation patterns. This may have a significant impact on the microbial communities present in the soil. However, the way these communities respond to seasonal variations in precipitation, particularly in the context of increased precipitation amounts, is not yet well understood.

**Methods:**

To explore this issue, a five-year (2012–2016) field study was conducted at the northeast boundary of the Ulan Buh Desert, examining the effects of increased precipitation during different periods of the growing season on both bacterial and fungal communities. The study included five precipitation pattern treatments: a control group (C), as well as groups receiving 50 and 100% of the local mean annual precipitation amount (145 mm) during either the early growing season (E50 and E100) or the late growing season (L50 and L100). The taxonomic composition of the soil bacterial and fungal communities was analyzed using Illumina sequencing.

**Results:**

After 5 years, the bacterial community composition had significantly changed in all treatment groups, with soil bacteria proving to be more sensitive to changes in precipitation timing than to increased precipitation amounts within the desert ecosystem. Specifically, the alpha diversity of bacterial communities in the late growing season plots (L50 and L100) decreased significantly, while no significant changes were observed in the early growing season plots (E50 and E100). In contrast, fungal community composition remained relatively stable in response to changes in precipitation patterns. Predictions of bacterial community function suggested that the potential functional taxa in the bacterial community associated with the cycling of carbon and nitrogen were significantly altered in the late growing season (L50 and L100).

**Discussion:**

These findings emphasize the importance of precipitation timing in regulating microbial communities and ecosystem functions in arid regions experiencing increased precipitation amounts.

## 1. Introduction

Climate change models have projected that the intensification of global hydrological cycles in terrestrial ecosystems will occur in the future, resulting from the increase in air temperature in past decades (Wetherald, 2009; Christensen et al., 2013; Marvel and Bonfils, 2013; Caspi et al., 2014; Sun et al., 2017). Changes in water circulation will cause alterations in precipitation patterns, such as precipitation amount and distribution (Piao et al., 2000; Austin et al., 2004; Clark et al., 2009; Delavaux et al., 2021). It is reported that the average precipitation amount over the mid-latitude land areas of the Northern Hemisphere and interannual variation in precipitation amounts will likely increase (Solomon et al., 2007). Additionally, changes in the distribution of precipitation have been observed worldwide, such as the shift of precipitation peak from summer to spring in the savannas of North Africa (Volder et al., 2013) and the tendency of decreased summer precipitation in Europe (Beier et al., 2012). However, our understanding of the ecological consequences of changes in precipitation distribution is only beginning to emerge.

The desert ecosystem constitutes nearly one-third of the Earth’s land surface (Julie Laity, 2009), and is characterized by low water availability, low nutrient levels, and low vegetation cover. Precipitation events largely drive soil ecological processes in this ecosystem since they largely affect soil water availability. Previous studies have found that increasing precipitation significantly stimulate soil respiration (Hsu et al., 2012) and aboveground net primary productivity (Xu et al., 2016), and alter nitrogen transformation (Song et al., 2020), plant phenology (Bao et al., 2021) in desert ecosystem, which potentially dampening future climate change (Knapp et al., 2008; Thomey et al., 2011; Hsu et al., 2012; Wang S. et al., 2018; Deng et al., 2021; Gao et al., 2021). Unlike other ecosystems, desert ecosystem experience conditions of chronic water shortage, water is the main limiting resource for biological activity in this area. Consequently, the desert is anticipated to be much more sensitive to precipitation pattern changes than other ecosystems (Maestre et al., 2016).

Soil bacterial and fungal communities are important components in desert ecosystem (Wagg et al., 2021). Since bacterial and fungal communities regulate the carbon cycling, nitrogen cycling, organic matter decomposition and nutrient acquisition, they are considered the dominant ecological drivers of desert ecosystem (Quoreshi et al., 2019). Bacterial and fungal communities are very sensitive to water availability (Rasche et al., 2011; Barnard et al., 2013). Thus, any change in precipitation patterns that alters soil microbial community characteristics can ultimately cause shifts in ecosystem functioning (Bell et al., 2014). Previous studies conducted in semi-arid grassland, agricultural and forest ecosystems have indicated that changes in precipitation amounts will directly affect microbial metabolism, structures, and compositions (Huang and Hall, 2017; Liu et al., 2022), which can potentially impact ecosystem functions. Nevertheless, compared with the above-mentioned ecosystems, fewer studies have been conducted on precipitation changes in the desert due to technology limitations and poor research conditions (Nielsen and Ball, 2015; Wang D. et al., 2018). In these sporadic studies, increased precipitation amounts were found to significantly alter the microbial community structure (Wang S. et al., 2018; Deng et al., 2021; Gao et al., 2021). Furthermore, changes in precipitation frequency and magnitude also affect the activity of soil microorganisms in these ecosystems (Bachar et al., 2012; Peng et al., 2013; Engelhardt et al., 2018; Gao et al., 2021).

Plant growth and primary productivity are known to be stimulated by increased precipitation amounts, as supported by various studies (Robertson et al., 2009; Bao et al., 2020; Cheng et al., 2021; Fan et al., 2021). However, the water requirements of plants vary during different developmental stages (Gutierrez et al., 2010; Alam et al., 2021; Sun H. et al., 2021), with higher water requirements in the early growing season than in the late growing season for optimal growth (Sun R. et al., 2021). Thus, the timing of precipitation in the context of increased precipitation amounts may play a crucial role in regulating plant growth. Additionally, changes in precipitation timing may also affect soil microbial communities, as plants are known to have an impact on these communities. Recent studies have noted that different seasonal distributions of precipitation can have profound effects on soil microbial communities in grassland, agricultural, and forest ecosystems (Wan et al., 2007; Parker and Schimel, 2011; Peng et al., 2013). Thus, to predict the responses of microbial community to increased precipitation under future climate scenarios, the different precipitation timing must be considered. However, few studies have examined the effects of increased precipitation amounts and timing on soil microbial communities in desert ecosystems (Huang and Hall, 2017; Liu et al., 2022).

According to many climate models predictions, the precipitation in desert areas of northwest China will increase under future climate change conditions (Gao et al., 2012; Chen, 2013; Li B. et al., 2016; Wang et al., 2017). Specifically, the annual precipitation in Northwest China is projected to increase by 50% at the end of the twenty-first century (Gao et al., 2012) and based on an RCP8.5 scenario, the annual increase in precipitation in some desert areas of northwest China will reach 100% or even higher than that at the end of the twentieth century (Gao et al., 2012). To address this research gap, we conducted a five-year (2012–2016) simulated field experiment in the Ulan Buh Desert of northern China by adding 0, 50, and 100% of local annual precipitation with two distribution patterns, only in the early or in the late growing season. Based on the growth cycle of *Nitraria tangutorum* in this ecosystem, the entire growing season could be divided into two periods (Bao et al., 2022). The main objectives of this study were to investigate: (1) How the timing and amount of precipitation influence the soil microbial community composition in the desert ecosystem; and (2) how the timing and amount of precipitation influence the soil microbial community functions. Global climate change is a pressing issue with far-reaching ecological implications, which not only includes the enhancement of temperature but also includes the alteration of precipitation patterns. Desert ecosystems are known to be highly sensitive to climate change, particularly in terms of altered precipitation patterns. By examining how changes in precipitation timing and amounts influence microbial communities, our research provides valuable insights into the potential consequences of global warming on desert ecosystem functioning.

## 2. Materials and methods

### 2.1. Study site

The study was conducted in a simulated precipitation increase platform, which locates at the northeastern edge of the Ulan Buh Desert, Dengkou County, Inner Mongolia, China (40°24’ N, 106°43′ E, 1,050 m a. s. l.), and is managed by the Institute of Desertification Studies, Chinese Academy of Forestry. The soil types at the study site are sandy and gray-brown desert soil (Cambic Arenosols and Luvic Gypsisols in FAO taxonomy). The site has an arid continental climate with an average annual precipitation of 145 mm (1978–2007) and a temperature of 7.8°C. About 77.5% of the annual precipitation occurs between June and September. The average annual potential evapotranspiration is 2,327 mm (Li et al., 2013).

The plant type is temperate desert shrubs with 20–30% vegetation cover. The dominant plant species in this ecosystem is *Nitraria tangutorum*. The plant community develops on nabkhas (a sand dune that forms around plant).

### 2.2. Experimental design

This long-term experiment was originally established in 2012 with a randomized full block partition, which was designed with four treatments and control. Each treatment and control have 4 replicates, for a total of 20 plots. The plots were separated by a buffer zone of 5 m or more to avoid mutual influence. A natural nabkha is centrally located within each plot. The height of the nabkhas ranged within 1.18–1.40 m, with a basal diameter of 5.75–8.83 m. Based on the growth cycle of general plants in this ecosystem, including *Nitraria tangutorum*, the entire growing season could be divided into two periods, early growing season (E, May 25 to July 10) and late growing season (L, July 25 to September 10), which were also designed for the two periods of precipitation increase (Supplementary Table S1). Based on the local annual average precipitation (145 mm), we designed two gradients of precipitation increase (50 and 100%). Thus, we selected nabkhas with similar growth conditions and applied control and four precipitation treatments: Control (C) = ambient precipitation, E50 = ambient precipitation+50% of the local annual average precipitation in the early growing reason, E100 = ambient precipitation +100% of the local annual average precipitation in the early growing reason, L50 = ambient precipitation +50% of the local annual average precipitation in the late growing reason, L100 = ambient precipitation +100% of the local annual average precipitation in the late growing reason. The water used in the experiment was pumped from a nearby well and stored in a tank. It was sprayed onto the land with an irrigation system installed on top of each nabkha. Since we used an irrigation system with two freely rotatable spray arms, the water could be evenly distributed over the treatment area (Supplementary Figure S1). To minimize water evaporation, water was added to the treatment area in the morning when the temperature was relatively low. The groundwater table is about 7.8 m (below 5 m), so plant growth is not affected by groundwater. In addition, the irrigation water and domestic water in surrounding areas were all from the Yellow River, and the underground water level is very stable, reflecting the water quality in this area is very consistent over time. An EM50 soil temperature and moisture measurement system (Decagon Devices, Pullman, United States) was installed in the sample plots for automatic monitoring of soil temperature and moisture.

### 2.3. Soil sampling and preparation

Soil samples were collected 15 days after the last increase in precipitation during the growing season (25th September 2016). To estimate the average variation in soil microbial communities across nabkhas, soil samples were selected within bare patches with similar environmental conditions in all nabkhas to reduce the effect of plant root distribution. In addition, topsoil (0–20 cm depth) was randomly collected from five representative points (four corners and one center) in each experimental area using a 5-cm diameter drill. The augers were cleaned with sterile water and air dried before each sample. Soil samples from the same test area were pooled to obtain a composite soil sample. The composite soil samples were packed in polyethylene bags and immediately stored in containers with ice packs for transferring the samples to the laboratory at low temperatures. The composite soil samples were sieved through a 2 mm screen to remove visible roots, residues, and stones. The samples were then divided into three parts: the first part was stored at −80°C for soil DNA extraction; the second part was stored at 4°C for measurement of soil soluble inorganic nitrogen, including nitrate (NO_3_^−^-N) and ammonium (NH_4_^+^-N). The third part was air dried to measure soil pH, total carbon (TC), total nitrogen (TN) and total phosphorus (TP). Soil samples were acquired at the fifth year of the water adding experiment, portraying a cumulative 5-year response to sustained precipitation manipulation.

### 2.4. Measurement of biotic and abiotic factors

Permanent plots of 0.5 m × 0.5 m were established in each plot to conduct a plant survey and record plant species and species richness. To eliminate as much as possible the effect of light differences in different directions on the growth of nabkha, a representative small sample plot of 0.5 m × 0.5 m in size was set up in the middle of the nabkha in the east–west and north–south directions of the nabkha, and a 10 cm × 10 cm mesh grid was used to determine the plant coverage within each plot and to investigate the plant species within each plot. Plant coverage was estimated visually for species that did not occur at junctions or for species that occurred at junctions but occupied small areas in the quadrats.

Long-term soil temperature and soil moisture dynamics at 20 cm depth in four typical plots within each treatment were automatically monitored every 30 min by an EM50 data logger system (Decagon, WA, United States), and values were averaged throughout the month. Soil pH was determined at a soil/water mass ratio of 1:2.5. Soil inorganic N (NH_4_^+^-N and NO_3_^−^-N) was extracted using 2 mol L ^−1^ KCl, and their contents were measured using continuous flow analytical system (SANN++ System, Skalar, Holland). The total carbon (TC) was measured by an elemental analyzer (FLASH 2000 CHNS/O), total nitrogen (TN) was determined by the Kjeldahl method, total phosphorus (TP) was measured by ammonium molybdate spectrophotometric method.

### 2.5. Soil DNA extraction and sequencing

Total DNA was extracted from 0.5 g sample of soil using an Ultra Clean^TM^ Soil DNA Isolation Kit (MO BIO Laboratories, Carlsbad, San Diego, United States) according to the manufacturer’s instructions, with the concentration (>100 ng μL^−1^) measured by a NanoDrop 2000 Spectrophotometer (Thermo Fisher Scientific, Carlsbad, United States). The DNA purity of each sample was A260/A280 ratio between 1.8 and 2.0, and A260/A230 ratio greater than 1.5.

To assess the taxonomic profiles of bacterial communities, the V4 region of the 16S rRNA gene was sequenced using Illumina HiSeq2500 platform (Caporaso et al., 2012) at Novogene Bioinformatics Technology Co., Ltd. (Beijing, China). The V4 hypervariable region of bacterial 16S rRNA gene was amplified with the barcoded primer set consisting of 515F (50-GTGCCAGCMGCCGCGGTAA-30) and 806R (50-GGACTACHVGGGTWTCTAAT-30). The ITS1 region of the fungal rRNA gene was amplified by the primers ITS5-1737F (50-GGAAGTAAAAGTCGTAACAAGG-30) and ITS2-2043R (50 -GCTGCGTTCTTCATCGATGC-30). All sequencing libraries involved in the experiments were generated using the TruSeq DNA PCR-Free Sample Preparation Kit (Illumina, San Diego, CA, United States) kit with index codes added in strict accordance with the manufacturer’s instruction manual recommendations. We assessed the quality of the libraries using on the Qubit 2.0 Fluorometer (Life Technologies, Carlsbad, CA, United States) and the Agilent Bioanalyzer 2,100 system (Agilent Technologies Inc., Santa Clara, CA, United States). Finally, we used the Illumina HiSeq2500 platform to sequence the libraries and perform genetic analysis of the 250 bp paired-end reads. Paired-end reads were tagged by their unique barcodes and thus assigned to samples. Then, these sequences were truncated by cutting off the barcode and primer sequences. We used FLASH (V1.2.7,^1^ accessed on 1 October 2017) to merge the paired-end reads and the QIIME (V1.7.0,^2^ accessed on 31 October 2017) platform (Caporaso et al., 2010) using default settings for quality filtering of the merged sequences. The filtered sequences were compared with the reference database (Gold database,^3^ accessed on 10 November 2017). The chimeric sequences are removed after they are detected using the UCHIME algorithm (UCHIME Algorithm,^4^ accessed on 25 October 2017) Subsequent sequence analysis was performed on the UPARSE (v7.0.1001,^5^ accessed on 1 December 2017) platform (Edgar, 2013). We considered sequences with 97% similarity to be considered as the same OTUs. We consider the most abundant sequence in each OTU as its representative sequence and annotate the taxonomic information of the representative sequence using the Silva database as the reference database. To make our predictions more informative, we used two methods to predict the potential function of the bacterial community. On the one hand we used the FAPROTAX database to predict the potential functional taxa of the bacterial community (Jewell et al., 2016). On the other hand, we used PICRUSt2 (Douglas et al., 2020) to predict the potential functional taxa of the bacterial community and annotated them against the KEGG (Kyoto Encyclopedia of Genes and Genomes) database (Kanehisa and Goto, 2000). And the putative taxonomic clusters of fungi were predicted by FUNGuild (Nguyen et al., 2016). Information related to sequence reads generated in this study was archived in the sequence read archive database of the National Center for Biotechnology Information under accession number PRJNA946553.

### 2.6. Statistical analysis

We used the species richness index, Shannon diversity index and Pielou’s evenness index, and Faith’s PD index to assess microbial alpha-diversity. To evaluate dissimilarities in microbial community composition among the different treatments, we employed analysis of similarity (ANOSIM) and permutational multivariate analysis of variance (Adonis). The results were visualized using Nonmetric multidimensional scaling analysis (NMDS). One-way ANOVA was conducted to compare the effects of different precipitation treatments at the phyla, order, family, and genus levels. *Post hoc* analyses were performed using Fisher’s least significant difference (LSD) test. Two-way ANOVA was used to examine the effects of increasing precipitation amount and time on microbial communities and environmental factors at the phyla, order, family, and genus levels. In order to comprehensively and extensively assess the influence of precipitation seasonality and amount on microbial taxa groups, we have selected the top 10 taxa at each taxonomic level as representatives, enabling a comparison of the diverse effects of precipitation on the microbial community. Normality of the data was verified using Shapiro–Wilk test. Bartlett’s test was used for homogeneity of variance test. Almost all the taxa group did not show normal distribution or conform to homogeneity of variance test. Thus, the nonparametric analysis, two-way permutational multivariate analysis of variance (PERMANOVA), was performed. Mantel test and spearman’s correlation coefficients were used to analyze the effects of plant/soil variables on the bacterial and fungal community compositions. Moreover, in order to further investigate the impact of environmental variables on microbial community diversity, we employed a random forest model to examine the correlations between bacterial and fungal diversities and environmental variables. All statistical analyses and figures described above were completed with R 4.0.0 (accessed on 10 April 2021)^6^ (R Core Team, 2021). The vegan package (Oksanen et al., 2018) was used for computing the Bray Curtis dissimilarity, the Mantel test, and the NMDS. Spearman rank correlations were computed by the R function cor with method = “spearman” (R Core Team, 2021), and adjusted all *p*-values by using the Benjamini and Hochberg false discovery rate (FDR) for multiple testing (Benjamini et al., 2006). The package pairwise.adonis was used for conducting the PERMANOVA (Martinez Arbizu, 2017). Package randomForests^7^ (Breiman, 2001) was used to perform random forest regression analysis. The ggplot2 package (Wickham, 2016) for generating the figures. Package pheatmap^8^ was used to generate the heat maps.

## 3. Results

### 3.1. Soil and plant variables under precipitation increase treatments in the early and late growing season

Our results showed that different precipitation treatments had a significant impact on soil physicochemical properties in the desert ecosystem (Supplementary Table S2). Compared to the control, soil moisture at 20 cm depth in E50 and E100 plots decreased by 25.2 and 15.4% while increased by 39.5 and 134.3% in L50 and L100 plots, respectively. Soil pH decreased significantly in the E100, L50 and L100 plots, while there were no significant changes in the E50 plots. In addition, soil nitrate concentration (NO_3_^−^-N) in the L100 plots was 1.5 times higher than that in the control plots, but no significant changes were observed in the other plots. The E100 and L100 plots had higher plant community cover than the other treatments, but due to the large coefficient of variation (Supplementary Table S3), the changes of plant cover were not significant. Total soil carbon (TC), total soil nitrogen (TN), total soil phosphorus (TP) and plant species did not show any significant differences among all treatments. Ammonium concentration (NH_4_^+^-N) was below the detection limit in all treatments.

To investigate the effects of treatments and their interactions on environmental factors, we performed two-way PERMANOVA analysis. Our results showed that increasing precipitation amount (“precipitation amounts” hereafter), different growing season periods (“precipitation season” hereafter), and their interactions (“interaction” hereafter) all had significant effects on soil moisture and soil temperature at 20 cm depth of soil (*p* < 0.001) (Supplementary Table S4). Precipitation season and interaction had significant effects on pH (*p* < 0.05) (Supplementary Table S4). Precipitation amounts individually had a significant effect on NO_3_^−^-N content (*p* < 0.05) (Supplementary Table S4).

### 3.2. Soil bacterial and fungal alpha-diversity and community composition under precipitation increase treatments in the early and late growing season

**Figure 1:**
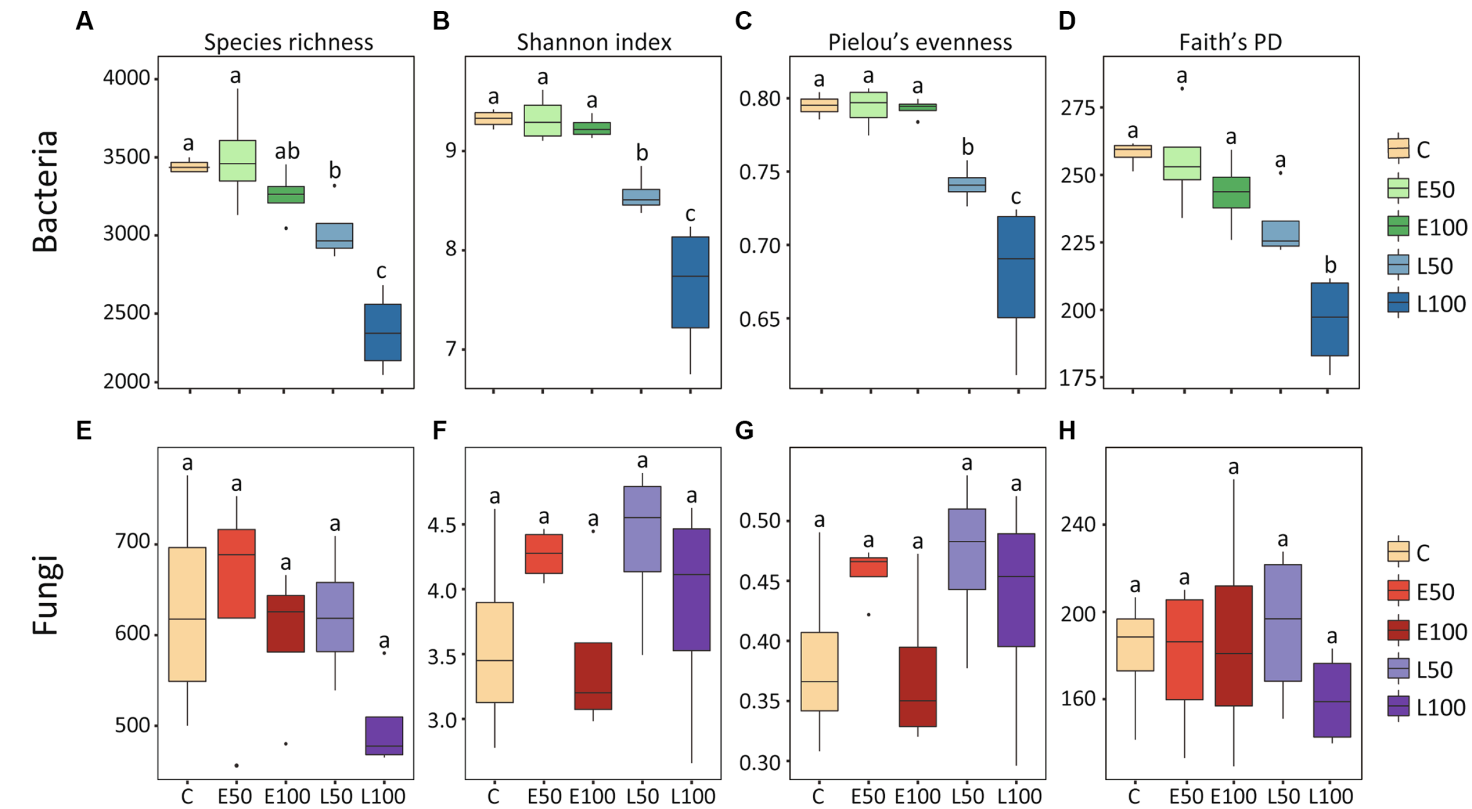
The alpha diversity of the bacterial community exhibited a declining trend, whereas the alpha diversity of the fungal community did not show significant changes. The alpha-diversity of bacterial **(A–D)** and fungal **(E–H)** taxonomic communities under different precipitation treatments are depicted in the box plots. The height of each box represents the range from the lower 1/4 quantile to the upper 1/4 quantile. Outliers are denoted as solid black circles positioned above or below the boxes. Lowercase letters (a, b, c) indicate significant differences across treatments, determined through ANOVA analysis, and *post hoc* analyses were conducted using Fisher’s least significant difference (LSD) test. Control (C) = ambient precipitation, E50 = ambient precipitation +50% of the local annual average precipitation in the early growing reason, E100 = ambient precipitation +100% of the local annual average precipitation in the early growing reason, L50 = ambient precipitation +50% of the local annual average precipitation in the late growing reason, L100 = ambient precipitation +100% of the local annual average precipitation in the late growing reason.

**Figure 2:**
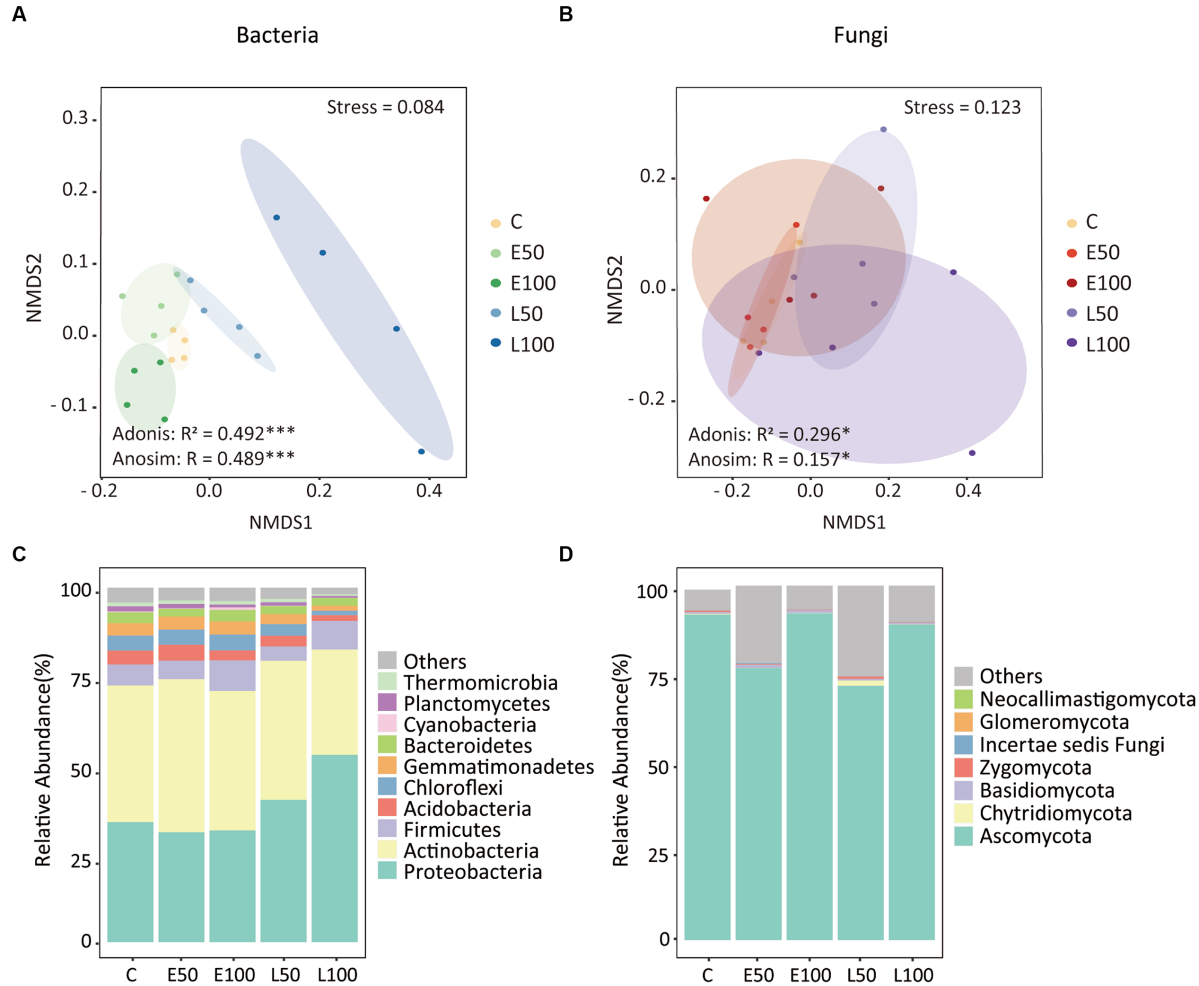
Nonmetric multidimensional scaling (NMDS) of Bray–Curtis’s dissimilarities highlighted that bacterial **(A)** and fungal **(B)** community structures were different among all treatments. Similarity values among the samples were examined via the Adonis and ANOSIM tests, which are shown in each plot. Ellipses in the plots denote 90% confidence intervals. The relative abundances of the dominant bacterial **(C)** and fungal **(D)** phyla in all treatments as shown. Further details regarding the differences in bacterial and fungal phyla among treatments can be found in Table 2. There were no significant changes in the relative abundances of fungal phyla.

To further investigate changes in soil microbial communities following precipitation, we conducted an analysis of the alpha-diversity of bacterial and fungal communities in the soil separately (Figure 1). Our findings indicate that an increase in precipitation during the early growing season (E50 and E100 plots) did not have a significant impact on bacterial species richness, Shannon diversity, Pielou’s evenness, and Faith’s PD. In contrast, an increase in precipitation during the late growing season led to a significant reduction in these bacterial alpha-diversity indices (Figures 1A–D), with the lowest indices observed in the L100 plots. Additionally, precipitation had no significant effect on fungal alpha-diversity in the early or late growing seasons (Figures 1E–H). Our NMDS results for bacterial communities showed that bacterial communities were clearly separated from each other (Figure 2A). This was confirmed by Adonis analysis (*R*^2^ = 0.492, *p* = 0.001) (Figure 2A). Although fungal communities could not be clearly separated from each other (Figure 2B), Adonis results still indicated that precipitation still had a statistically significant effect on fungal communities during different growing seasons (R^2^ = 0.296, *p* = 0.032) (Figure 2B).

Two-way PERMANOVA analysis showed that although both precipitation season and precipitation amounts had significant effects on the bacterial alpha-diversity indexes. However, compared to the precipitation amount, the precipitation season appeared to have a stronger effect (precipitation seasons: *p* < 0.001 vs. precipitation amount: *p* > 0.006) (Table 1). On the other hand, we observed that neither precipitation season, nor precipitation amounts, nor their interactions had significant effects on the fungal alpha-diversity indices.

**Table 1:** Results of two-way PERMANOVA on the effects of season (S), amounts (A) and their interactions (S × A) on the alpha diversity indices and communities.

		S	A	S × A
		Pr(>F)	Pr(>F)	Pr(>F)
Bacteria	Species richness	<0.001***	0.006**	0.115
	Shannon diversity	<0.001***	0.014*	0.041*
	Pielou’s evenness	<0.001***	0.033*	0.060.
	Faith’s PD	<0.001***	0.015*	0.184
	Community	<0.001***	0.354	0.263
Fungi	Species richness	0.184	0.079.	0.443
	Shannon diversity	0.412	0.065.	0.629
	Pielou’s evenness	0.321	0.094.	0.526
	Faith’s PD	0.564	0.755	0.295
	Community	0.099.	0.213	0.491

*n* = 4. S, Precipitation seasonality; A, amounts of precipitation; S × A, Interaction between precipitation seasonality and precipitation amounts. represents 0.05 < *p* < 0.1; *represents 0.01 < *p* < 0.05; **represents 0.001 < *p* < 0.001; ***represents *p* < 0.001.

The phyla of Proteobacteria (37.9%), Actinobacteria (38.0%), Firmicutes (6.4%), Acidobacteria (3.2%), Chloroflexi (3.5%), Gemmatimonadetes (3.0%), and Bacteroidetes (2.6%) were the dominant phyla in our site, making up over 90% of the total relative abundance (Figure 2C). Specifically, Proteobacteria increased from 33.9 ± 1.2% in control to 52.9 ± 11.3% in L100 plots (Table 2). However, there was no significant change in Proteobacteria abundance in E50 and E100 plots (Table 2). In contrast, the relative abundance of Acidobacteria significantly decreased from 3.9 ± 0.8% in control to 1.6 ± 0.5% in L100 plot. Chloroflexi decreased significantly from 4.3 ± 0.6% in control plots to 1.3 ± 0.2% in L100 plot and Gemmatimonadetes from 3.5 ± 0.4% in control plot to 1.4 ± 1.2% in L100 plot. At the order level, four of 10 most abundant orders demonstrated significantly increases in L100 plots, including Methylophilales, Rhizobiales, Rhodobacterales, and Clostridiales whereas two orders demonstrated significantly decreases, including Bacillales and Solirubrobacterales (Table 2). At the family level, three of 10 most abundant families demonstrated significantly increases in L100 plots, including *Methylophilaceae*, *Methylobacteriaceae*, *Rhodobacteraceae* whereas only the family of *Bacillaceae* decreased (Table 2). At the genus level, only three abundant genera demonstrated increases in L100 plots, including *Methylobacillus*, *Methylobacterium*, and *Paracocccus* whereas two genera demonstrated decreases, including *Bacillus* and *Rubellimicrobium* (Table 2). Moreover, the results of the two-way PERMANOVA showed that precipitation seasons had significant effects on seven of the 10 most abundant phyla, including Proteobacteria, Acidobacteria, Chloroflexi, Gemmatimonadetes, Bacteroidetes, Cyanobacteria, and Thermomicrobia (Table 3). Precipitation amounts significantly affected five of these phyla, including Acidobacteria, Chloroflexi, Bacteroidetes, Planctomycetes, and Thermomicrobia. At the finer level, precipitation seasons had significant effects on four of the 10 most abundant order (Rhizobiales, Bacillales, Solirubrobacterales, and Rhodobacterales), three of the 10 most abundant families (*Methylobacteriaceae*, *Rhodobacteraceae*, and *Bacillaceae*) and four of the ten most abundant genera (*Methylobacterium*, *Paracocccus*, Bacillus, and *Rubellimicrobium*), whereas precipitation amount had significant effects on only one family (*Streptomycetaceae*) and one genus (*Microvirga*) among most abundant taxa (Table 3). Thus, although both precipitation seasons and precipitation amount had significant effects on the bacterial community compositions, the precipitation season had a stronger effect compared to precipitation amounts (Table 3).

**Table 2:** Effects of precipitation on the relative abundances of the top 10 soil bacterial and fungal phyla, orders, families, and genus (*n* = 4).

	Level	Change	Taxa groups	C (%)	E50 (%)	E100 (%)	L50 (%)	L100 (%)
Bacteria	Phylum	Increased	Proteobacteria	33.91 ± 1.17b	31.05 ± 3.05b	31.55 ± 3.32b	40.17 ± 8.77b	52.88 ± 11.28a
			Firmicutes	5.92 ± 1.20a	5.19 ± 1.38a	8.61 ± 3.67a	4.07 ± 0.71a	8.06 ± 6.33a
		Decreased	Actinobacteria	38.52 ± 1.30ab	43.19 ± 3.49a	39.33 ± 3.27a	39.23 ± 6.73a	29.70 ± 11.39b
			Acidobacteria	3.90 ± 0.76ab	4.50 ± 1.16a	2.85 ± 0.32b	2.99 ± 0.49b	1.60 ± 0.50c
			Chloroflexi	4.27 ± 0.62a	4.32 ± 0.71a	4.46 ± 0.35a	3.27 ± 0.14b	1.28 ± 0.19c
			Gemmatimonadetes	3.50 ± 0.42a	3.66 ± 0.19a	3.67 ± 0.73a	2.83 ± 0.60a	1.40 ± 1.17b
			Bacteroidetes	3.08 ± 0.66a	2.16 ± 0.32b	3.30 ± 0.53a	2.27 ± 0.32b	2.22 ± 0.44b
			Cyanobacteria	0.25 ± 0.05ab	0.15 ± 0.02ab	0.68 ± 0.81a	0.15 ± 0.03ab	0.05 ± 0.02b
			Planctomycetes	1.39 ± 0.42a	1.24 ± 0.26ab	0.78 ± 0.37bc	0.95 ± 0.39abc	0.47 ± 0.23c
			Thermomicrobia	1.11 ± 0.28a	0.96 ± 0.17a	1.01 ± 0.14a	0.96 ± 0.15a	0.46 ± 0.11b
	Order	Increased	Methylophilales	1.50 ± 0.68b	1.73 ± 0.59b	0.62 ± 0.12b	4.92 ± 4.44ab	12.10 ± 12.95a
			Rhizobiales	9.61 ± 0.53b	8.63 ± 1.49b	8.67 ± 0.79b	11.96 ± 1.53b	18.02 ± 6.02a
			Rhodobacterales	4.19 ± 0.61b	3.56 ± 0.95b	4.15 ± 0.71b	6.87 ± 2.46a	6.80 ± 2.51a
			Clostridiales	0.10 ± 0.03b	0.14 ± 0.09b	0.38 ± 0.39b	0.09 ± 0.02b	3.53 ± 3.38a
		Decreased	Bacillales	5.70 ± 1.10ab	4.99 ± 1.32b	7.86 ± 3.08a	3.87 ± 0.65bc	2.49 ± 0.62c
			Solirubrobacterales	6.13 ± 0.43a	6.59 ± 0.92a	6.91 ± 2.32a	5.16 ± 0.46a	2.22 ± 0.72b
			Frankiales	4.60 ± 0.41ab	5.10 ± 0.81ab	6.05 ± 1.00a	4.94 ± 0.60ab	4.15 ± 1.80b
		Unchanged	Micrococcales	9.53 ± 1.81a	9.85 ± 3.52a	8.12 ± 2.02a	11.87 ± 5.65a	10.95 ± 4.83a
			Propionibacteriales	5.11 ± 0.47a	6.68 ± 0.94a	4.14 ± 0.63a	6.09 ± 0.97a	6.61 ± 3.96a
			Sphingomonadales	5.21 ± 0.43a	4.65 ± 0.33a	4.82 ± 0.46a	5.19 ± 1.28a	5.43 ± 0.50a
	Family	Increased	*Methylophilaceae*	1.50 ± 0.68b	1.73 ± 0.59b	0.62 ± 0.12b	4.92 ± 4.44ab	12.10 ± 12.95a
			*Methylobacteriaceae*	4.05 ± 0.47b	3.19 ± 0.81b	3.58 ± 0.58b	6.55 ± 1.73ab	10.94 ± 6.55a
			*Rhodobacteraceae*	4.19 ± 0.61b	3.56 ± 0.95b	4.15 ± 0.71b	6.87 ± 2.46a	6.80 ± 2.51a
			*Sphingomonadaceae*	2.82 ± 0.26ab	2.74 ± 0.29ab	2.51 ± 0.37b	3.04 ± 1.06ab	3.43 ± 0.51a
		Decreased	*Bacillaceae*	2.59 ± 0.55ab	2.25 ± 0.86bc	3.65 ± 1.51a	1.94 ± 0.26bc	1.14 ± 0.37c
			*Streptomycetaceae*	0.98 ± 0.25ab	1.74 ± 1.02a	0.82 ± 0.09b	0.94 ± 0.14b	0.50 ± 0.40b
		Unchanged	*Micrococcaceae*	8.02 ± 1.83a	8.29 ± 3.36a	6.52 ± 1.71a	10.27 ± 5.57a	9.01 ± 4.38a
			*Nocardioidaceae*	5.02 ± 0.46a	6.56 ± 0.88a	4.02 ± 0.60a	6.00 ± 0.96a	6.50 ± 3.87a
			*Geodermatophilaceae*	3.45 ± 0.39a	3.69 ± 0.64a	4.49 ± 0.79a	3.69 ± 0.47a	3.37 ± 1.52a
			*Lactobacillaceae*	0.04 ± 0.03a	0.01 ± 0.01a	0.12 ± 0.16a	0.03 ± 0.03a	1.18 ± 2.23a
	Genus	Increased	*Methylobacillus*	1.02 ± 0.46b	1.26 ± 0.43b	0.49 ± 0.09b	3.42 ± 3.41ab	9.86 ± 11.00a
			*Methylobacterium*	1.36 ± 0.37b	1.25 ± 0.41b	0.54 ± 0.07b	4.18 ± 1.67ab	8.31 ± 6.51a
			*Nocardioides*	3.35 ± 0.38ab	4.29 ± 0.20ab	2.68 ± 0.53b	4.31 ± 0.90ab	5.36 ± 3.32a
			*Paracocccus*	2.02 ± 0.37b	1.70 ± 0.63b	1.42 ± 0.49b	4.86 ± 2.29a	5.44 ± 2.19a
			*Sphingomonas*	2.76 ± 0.26ab	2.67 ± 0.28ab	2.40 ± 0.38b	2.99 ± 1.04ab	3.33 ± 0.56a
		Decreased	*Bacillus*	1.39 ± 0.36ab	1.22 ± 0.42bc	2.19 ± 1.20a	0.94 ± 0.22bc	0.42 ± 0.15c
			*Microvirga*	2.51 ± 0.15ab	1.81 ± 0.40c	2.89 ± 0.61a	2.20 ± 0.21bc	2.40 ± 0.61abc
			*Rubellimicrobium*	1.80 ± 0.25ab	1.57 ± 0.33bc	2.39 ± 0.71a	1.65 ± 0.24bc	1.07 ± 0.26c
		Unchanged	*Arthrobacter*	4.79 ± 1.31a	4.54 ± 1.45a	4.49 ± 1.11a	4.40 ± 0.78a	5.01 ± 0.87a
			*Lactobacillus*	0.04 ± 0.03a	0.01 ± 0.01a	0.12 ± 0.16a	0.03 ± 0.03a	1.18 ± 2.23a
Fungi	Phylum	Unchanged	Ascomycota	91.863 ± 2.69a	76.781 ± 13.605a	92.204 ± 4.693a	71.75 ± 21.277a	89.045 ± 7.852a
			Chytridiomycota	0.18 ± 0.266a	0.038 ± 0.023a	0.09 ± 0.145a	1.493 ± 2.904a	0.074 ± 0.043a
			Basidiomycota	0.528 ± 0.24a	0.861 ± 0.776a	0.786 ± 0.304a	0.483 ± 0.073a	0.443 ± 0.124a
			Zygomycota	0.36 ± 0.306a	0.22 ± 0.246a	0.134 ± 0.06a	0.595 ± 0.47a	0.167 ± 0.1a
			Incertae sedis fungi	0.083 ± 0.047a	0.204 ± 0.285a	0.026 ± 0.014a	0.069 ± 0.047a	0.046 ± 0.044a
			Glomeromycota	0.02 ± 0.02a	0.009 ± 0.009a	0.009 ± 0.004a	0.006 ± 0.003a	0.004 ± 0.004a
			Neocallimastigomycota	0.001 ± 0.001a	0.0 ± 0.0a	0.0 ± 0.0a	0.002 ± 0.002a	0.0 ± 0.0a
	Order	Decreased	Hypocreales	60.86 ± 15.23a	47.95 ± 15.70ab	63.96 ± 18.27a	34.09 ± 10.86b	56.28 ± 22.32ab
			Eurotiales	5.69 ± 3.91a	3.02 ± 2.08ab	3.43 ± 4.82ab	1.67 ± 0.39ab	1.25 ± 0.38b
		Unchanged	Pleosporales	9.10 ± 4.31a	10.01 ± 9.26a	13.64 ± 6.75a	12.26 ± 4.94a	11.26 ± 6.74a
			Chaetothyriales	0.15 ± 0.07a	0.45 ± 0.47a	0.08 ± 0.02a	6.12 ± 8.13a	4.32 ± 5.36a
			Sordariales	6.32 ± 5.90a	6.28 ± 3.65a	4.12 ± 2.28a	7.29 ± 1.66a	2.44 ± 1.12a
			Botryosphaeriales	2.20 ± 3.50a	0.77 ± 0.99a	0.33 ± 0.12a	1.13 ± 1.13a	3.52 ± 6.25a
			Dothideales	0.07 ± 0.05a	0.17 ± 0.18a	0.05 ± 0.04a	0.06 ± 0.01a	1.69 ± 2.83a
			Rhizophlyctidales	0.16 ± 0.26a	0.02 ± 0.01a	0.07 ± 0.13a	1.48 ± 2.90a	0.01 ± 0.02a
			Microascales	1.37 ± 0.43a	1.63 ± 0.75a	0.94 ± 0.52a	1.90 ± 2.58a	1.90 ± 2.47a
			Incertae sedis Dothideomycetes	1.91 ± 2.18a	1.01 ± 0.26a	1.18 ± 0.71a	1.72 ± 0.83a	1.00 ± 0.83a
	Family	Increased	*Incertae sedis Hypocreales*	2.64 ± 0.61b	3.53 ± 2.28b	2.47 ± 0.96b	3.49 ± 1.97b	21.57 ± 18.64a
		Decreased	*Nectriaceae*	57.04 ± 15.25a	43.46 ± 17.43ab	57.33 ± 18.11a	28.97 ± 8.45b	32.13 ± 28.12ab
			*Chaetomiaceae*	6.00 ± 5.81ab	4.09 ± 0.88ab	3.88 ± 2.30ab	6.69 ± 1.50a	1.94 ± 0.99b
			*Trichocomaceae*	5.69 ± 3.91a	3.02 ± 2.08ab	3.43 ± 4.82ab	1.67 ± 0.39ab	1.25 ± 0.38b
		Unchanged	*Herpotrichiellaceae*	0.14 ± 0.07a	0.45 ± 0.47a	0.07 ± 0.01a	6.12 ± 8.13a	4.32 ± 5.36a
			*Pleosporaceae*	2.60 ± 0.94a	3.76 ± 1.10a	5.39 ± 3.14a	2.56 ± 1.09a	5.56 ± 5.08a
			*Incertae sedis Botryosphaeriales*	2.20 ± 3.50a	0.77 ± 0.99a	0.33 ± 0.12a	1.13 ± 1.13a	3.52 ± 6.25a
			*Sporormiaceae*	1.89 ± 2.63a	0.55 ± 0.37a	4.65 ± 5.58a	0.65 ± 0.48a	1.32 ± 1.33a
			*Bionectriaceae*	0.69 ± 0.53a	0.69 ± 0.53a	3.00 ± 4.81a	1.42 ± 2.26a	1.01 ± 0.71a
			*Testudinaceae*	2.07 ± 3.65a	0.05 ± 0.02a	0.24 ± 0.02a	0.14 ± 0.25a	0.02 ± 0.02a
	Genus	Increased	*Stachybotrys*	1.55 ± 0.39b	1.62 ± 1.21b	1.71 ± 1.10b	2.43 ± 1.70b	6.98 ± 4.75a
		Decreased	*Gibberella*	54.06 ± 15.23a	34.37 ± 13.90ab	53.39 ± 17.42ab	27.15 ± 7.83b	31.19 ± 27.62ab
			*Fusarium*	2.85 ± 1.33ab	9.01 ± 11.62a	3.88 ± 2.29ab	1.75 ± 0.71ab	0.80 ± 0.55b
		Unchanged	*Phialophora*	0.13 ± 0.07a	0.44 ± 0.47a	0.07 ± 0.01a	6.12 ± 8.13a	4.32 ± 5.36a
			*Camarosporium*	2.20 ± 3.50a	0.77 ± 0.99a	0.33 ± 0.12a	1.13 ± 1.13a	3.52 ± 6.25a
			*Alternaria*	2.32 ± 1.01a	3.36 ± 0.85a	4.96 ± 2.98a	2.06 ± 0.78a	5.29 ± 5.05a
			*Chaetomium*	3.70 ± 4.89a	0.98 ± 0.40a	1.22 ± 0.42a	1.76 ± 0.62a	0.89 ± 0.46a
			*Hydropisphaera*	0.68 ± 0.54a	0.68 ± 0.52a	2.98 ± 4.82a	1.41 ± 2.26a	1.01 ± 0.71a
			*Lepidosphaeria*	2.07 ± 3.65a	0.05 ± 0.02a	0.24 ± 0.02a	0.14 ± 0.25a	0.02 ± 0.02a
			*Aureobasidium*	0.07 ± 0.05a	0.17 ± 0.18a	0.04 ± 0.04a	0.06 ± 0.01a	1.69 ± 2.82a

Lowercase letters denote statistically significant differences (*p* < 0.05).

**Table 3:** Results of two-way PERMANOVA on the effects of season (S), amount (A) and their interactions (S × A) on the relative abundance of top 10 groups at phyla, order, family, and genus levels.

	Level	Taxa groups	S	A	S × A
			Pr(> F)	Pr(> F)	Pr(> F)
Bacteria	Phylum	Proteobacteria	0.002**	0.087.	0.116
		Actinobacteria	0.094.	0.101	0.461
		Firmicutes	0.666	0.064.	0.866
		Acidobacteria	0.003**	0.001***	0.734
		Chloroflexi	0.001***	0.003**	0.001***
		Gemmatimonadetes	0.001***	0.084.	0.087
		Bacteroidetes	0.033*	0.018*	0.011*
		Cyanobacteria	0.002**	0.418	0.003**
		Planctomycetes	0.105	0.016*	0.985
		Thermomicrobia	0.004**	0.014*	0.007**
	Order	Methylophilales	0.053.	0.393	0.25
		Rhizobiales	0.002**	0.082.	0.086.
		Micrococcales	0.275	0.542	0.852
		Bacillales	0.003**	0.405	0.031*
		Propionibacteriales	0.393	0.358	0.175
		Solirubrobacterales	0.001***	0.071.	0.029*
		Rhodobacterales	0.007**	0.779	0.729
		Clostridiales	0.094.	0.051.	0.084.
		Frankiales	0.098.	0.894	0.155
		Sphingomonadales	0.145	0.589	0.927
	Family	*Methylophilaceae*	0.053.	0.393	0.25
		*Methylobacteriaceae*	0.009**	0.187	0.265
		*Micrococcaceae*	0.287	0.465	0.902
		*Nocardioidaceae*	0.368	0.344	0.168
		*Rhodobacteraceae*	0.007**	0.779	0.729
		*Geodermatophilaceae*	0.256	0.621	0.256
		*Bacillaceae*	0.008**	0.515	0.03*
		*Lactobacillaceae*	0.355	0.285	0.374
		*Sphingomonadaceae*	0.077.	0.796	0.352
		*Streptomycetaceae*	0.067.	0.031*	0.395
	Genus	*Methylobacillus*	0.069.	0.345	0.235
		*Methylobacterium*	0.008**	0.33	0.176
		*Nocardioides*	0.148	0.754	0.152
		*Paracocccus*	0.001***	0.852	0.608
		*Arthrobacter*	0.735	0.616	0.554
		*Lactobacillus*	0.353	0.285	0.373
		*Sphingomonas*	0.075.	0.908	0.361
		*Bacillus*	0.008**	0.492	0.041*
		*Microvirga*	0.856	0.022*	0.096.
		*Rubellimicrobium*	0.014*	0.601	0.007**
Fungi	Phylum	Ascomycota	0.552	0.021*	0.887
		Chytridiomycota	0.517	0.851	0.431
		Basidiomycota	0.121	0.841	0.925
		Zygomycota	0.161	0.057	0.243
		Incertae sedis fungi	0.699	0.092.	0.328
		Glomeromycota	0.094.	0.666	0.655
		Neocallimastigomycota	0.208	0.208	0.208
	Order	Hypocreales	0.237	0.047*	0.727
		Pleosporales	0.986	0.719	0.526
		Chaetothyriales	0.065.	0.664	0.775
		Sordariales	0.781	0.012*	0.28
		Botryosphaeriales	0.291	0.555	0.396
		Eurotiales	0.208	0.998	0.759
		Dothideales	0.299	0.308	0.237
		Rhizophlyctidales	0.354	0.349	0.315
		Microascales	0.517	0.715	0.715
		Incertae sedis Dothideomycetes	0.464	0.449	0.223
	Family	*Nectriaceae*	0.062.	0.396	0.59
		*Incertae sedis Hypocreales*	0.067.	0.097.	0.066.
		*Herpotrichiellaceae*	0.065.	0.663	0.775
		*Chaetomiaceae*	0.678	0.007**	0.012*
		*Pleosporaceae*	0.743	0.16	0.663
		*Incertae sedis Botryosphaeriales*	0.291	0.555	0.396
		*Sporormiaceae*	0.284	0.124	0.258
		*Trichocomaceae*	0.208	0.998	0.759
		*Bionectriaceae*	0.65	0.494	0.334
		*Testudinaceae*	0.315	0.582	0.033*
	Genus	*Gibberella*	0.131	0.229	0.426
		*Fusarium*	0.107	0.326	0.495
		*Phialophora*	0.065.	0.663	0.775
		*Stachybotrys*	0.041*	0.106	0.118
		*Camarosporium*	0.291	0.555	0.396
		*Alternaria*	0.75	0.132	0.597
		*Chaetomium*	0.359	0.218	0.04*
		*Hydropisphaera*	0.653	0.495	0.336
		*Lepidosphaeria*	0.315	0.582	0.033*
		*Aureobasidium*	0.299	0.308	0.237

*n* = 4. S, Precipitation seasonality; A, Amount of precipitation; S × A, Interaction between precipitation seasonality and precipitation amount; represents 0.05 < *p* < 0.1; *represents 0.01 < *p* < 0.05; ** represents 0.001 < *p* < 0.01; *** represents *p* < 0.001.

The fungal group Ascomycota accounted for an overwhelming majority (84.3%), followed by Chytridiomycota (0.4%), Basidiomycota (0.6%), Zygomycota (0.3%), and Incertae sedis fungi (0.1%) (Figure 2D). Approximately 14.3% of the sequences were unclassified. Furthermore, results of the one-way ANOVA and the two-way PERMANOVA showed that precipitation pattern change had no significant effect on the change in the relative abundance of the dominant fungal phyla (Tables 2–3). At the order level, the order of Hypocreales significantly decreased in L50 plot while the order of Eurotiales significantly decreased in L100 plot (Table 2). At the family level, the family of *Trichocomaceae* significantly decreases in L100 plots while only the family of *Incertae sedis Hypocreales* increased (Table 2). At the genus level, only *Stachybotrys* significantly increased in L100 plots whereas two genera demonstrated decreases, including *Gibberella* and *Fusarium* (Table 2). Moreover, the results of the two-way PERMANOVA showed that precipitation amount had significant effects on only one of the 10 most abundant phyla, which is Ascomycota (Table 3). Similarly, precipitation amount had significant effects on two of the 10 most abundant order (Hypocreales, Sordariales), one of the 10 most abundant families (*Chaetomiaceae*), whereas precipitation season had significant effects on only one genus (*Stachybotrys*) among most abundant taxa.

### 3.3. Relationships between bacterial and fungal communities and plant/soil properties at different precipitation patterns

**Figure 3:**
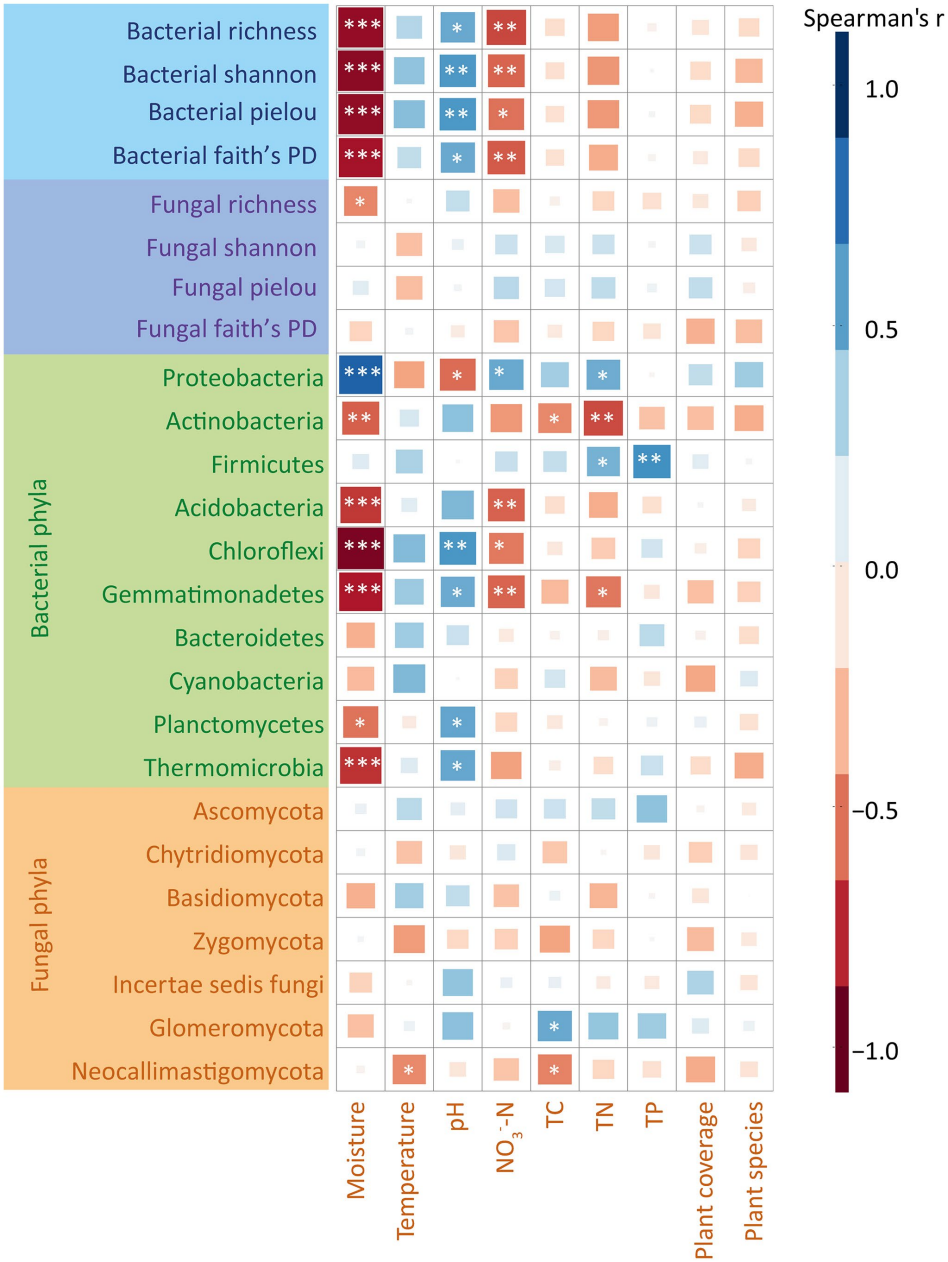
Spearman’s correlation coefficient analysis of bacterial and fungal communities and alpha diversity indices with environmental factors. The colors represent the magnitude of Spearman’s correlation coefficient r. Darker colors represent larger values of |r|, the more correlated. *r* > 0, representing positive correlation, is marked in blue. *r* < 0, representing negative correlation, is marked in red. ns represents *p* > 0.05; *represents 0.01 < *p* < 0.05; **represents 0.001 < *p* < 0.01; ***represents *p* < 0.001. TC, total carbon; TN, total nitrogen; TP, total phosphorus.

We utilized Mantel tests to assess the correlations between environmental variables and bacterial and fungal communities (Supplementary Figure S2). The results showed that at soil moisture at 20 cm depth, TN and NO_3_^−^-N significantly correlated with the composition of soil bacterial and fungal communities, while other environmental factors exhibited no significant correlation (Supplementary Figure S2). The random forest results showed that the alpha diversity of bacterial communities was significantly affected by moisture, pH and NO_3_^−^-N (Supplementary Figure S3), whereas no significant associations were observed between environmental factors and the alpha diversity of fungal communities. Additionally, to explore the correlations between each taxon and environmental factors in depth, we calculated Spearman’s correlation coefficients between each environmental variable with alpha indices of bacterial and fungal communities at the phylum level (Figure 3). On one hand, our results showed that soil moisture and NO_3_^−^-N were significantly and positively correlated with soil bacterial alpha index. The value of pH was significantly and negatively correlated with soil bacterial alpha index. The other environmental factors were not significantly correlated with the composition of soil bacterial and fungal communities. On the other hand, our results showed that moisture was significantly and positively correlated with the abundance of Proteobacteria (*r* = 0.75, *p* < 0.05). In contrast, moisture negatively correlated with the relative abundances of Acidobacteria (*r* = −0.68, *p* < 0.05), Chloroflexi (*r* = −0.76, *p* < 0.05) and Gemmatimonadetes (*r* = −0.69, *p* < 0.05) (Figure 3). Moreover, pH negatively correlated with the relative abundances of Proteobacteria (*r* = −0.68, *p* < 0.05), while positively correlated with Chloroflexi (*r* = 0.69, *p* < 0.05) (Figure 3). Regarding fungi, our results showed no significant correlations between any of the fungal phyla and the environmental factors (Figure 3).

### 3.4. Precipitation significantly altered the functions of microbial communities

**Figure 4:**
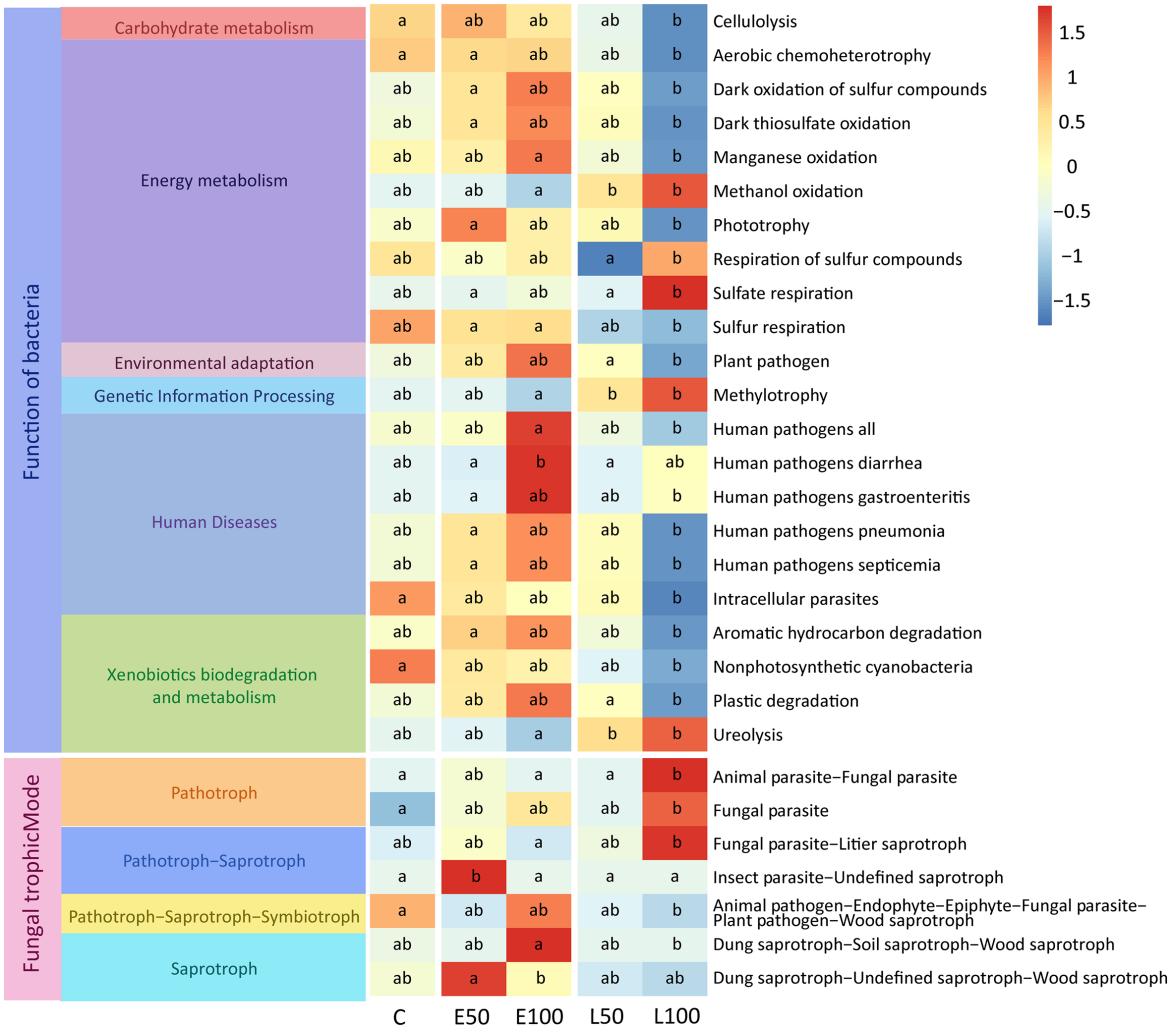
Potential functional taxa with significant variation in bacterial and fungal communities (*p* < 0.05). Our methods for predicting the potential function of soil bacterial and fungal communities are FAPROTAX and FUNGuild. Lowercase letters (a, b, c) within panels indicate significant differences among the treatments (*p* < 0.05). These differences were determined using a multiple-comparison test following ANOVA analysis, and *post hoc* analyses were conducted using Fisher’s least significant difference (LSD) test (*p* < 0.05).

In order to investigate the impact of changes in precipitation patterns on the functions of microbial communities in desert ecosystems, we utilized FAPROTAX to predict the potential ecological functions of soil bacterial community and use FUNGuild to predict the potential functions of fungal communities. The results of FAPROTAX revealed that some functional communities related to nutrient cycling changed significantly under different precipitation treatments (*p* < 0.05) in the soil bacterial community. Specifically, the functions related to organic synthesis (e.g., methylotrophy and phototrophy) were significantly enriched in the early growing season treatment plots (E50, E100) compared to control plots (*p* < 0.05) (Figure 4). Conversely, functions related to organic matter degradation (e.g., cellulolysis and ureolysis) were significantly inhibited in the late growing season treatment plots (L50, L100) compared to control plots (*p* < 0.05) (Figure 4). In addition, some potential functions related to sulfur respiration (e.g., respiration of sulfur compounds and sulfate respiration) were significantly reduced with increasing precipitation amount in the late growing season. Results from FUNGuild analysis demonstrated that the functions related to saprotroph (e.g., dung saprotroph−soil saprotroph−wood saprotroph) were significantly enriched in the early growing seasons treatment (E50, E100) plots compared to control plots (*p* < 0.05) (Figure 4). Conversely, functions related to pathotroph (e.g., fungal parasite) were significantly inhibited in the late growing season treatment plots (L100) compared to control plots (*p* < 0.05) (Figure 4). However, there were no significant changes in the functional taxa of fungi at the trophic Mode level (Supplementary Figure S4).

In order to make a more comprehensive prediction of potential functional taxa of bacterial communities, we also used PICRUSt2 software to predict bacterial communities, using the KEGG database comparison. The results of the comparison were 7,564 KOs, and functional proteins with average relative abundance less than 0.1% were filtered out, and 91 functional proteins were selected for the subsequent significance analysis. Finally, there were 39 functional proteins with significant changes (Supplementary Table S5). Consistent with the FAPROTAX results, most of the potential functional taxa with significant changes were from carbohydrate metabolism, lipid metabolism and cellular processes.

The effects of precipitation season and precipitation amount on the potential functions of bacterial and fungal communities were further analyzed using a two-way PERMANOVA analysis (Table 4). Our results showed that precipitation season had an impact on the potential functions of bacterial communities related to nutrient cycling include organic synthesis (e.g., methylotrophy and phototrophy) and organic matter degradation (e.g., cellulolysis and ureolysis) (Table 4). Precipitation amounts significantly affected the potential functions of bacterial communities related to sulfur respiration and respiration of sulfur compounds (Table 4). Although both precipitation seasons and precipitation amount had significant effects on the potential functional taxa of bacteria, compared with the precipitation amounts, the precipitation season seems to have a stronger effect than precipitation amount (Table 4). Furthermore, precipitation season, precipitation amount and their interaction had a significant effect on the potential Insect Parasite-Undefined Saprotroph function in the fungal community (Table 4).

**Table 4:** Results of two-way ANOVA on the effects of season (S), amount (A) and their interactions (S × A) on the potential functions with significant variation in bacterial and fungal communities.

	Functional groups	S	A	S × A
		Pr(>F)	Pr(>F)	Pr(>F)
Bacteria	Aerobic chemoheterotrophy	0.004**	0.244	0.232
	Aromatic hydrocarbon degradation	0.009**	0.505	0.186
	Cellulolysis	0.006**	0.13	0.554
	Dark oxidation of sulfur compounds	0.103	0.728	0.249
	Dark thiosulfate oxidation	0.081.	0.598	0.189
	Human pathogens all	0.010**	0.319	0.027*
	Human pathogens diarrhea	0.24	0.049*	0.237
	Human pathogens gastroenteritis	0.221	0.051.	0.219
	Human pathogens pneumonia	0.083.	0.593	0.194
	Human pathogens septicemia	0.082.	0.59	0.193
	Intracellular parasites	0.001**	<0.001***	0.013*
	Manganese oxidation	0.013*	0.854	0.061.
	Methanol oxidation	0.003**	0.547	0.155
	Methylotrophy	0.003**	0.537	0.148
	Nonphotosynthetic cyanobacteria	0.029*	0.392	0.604
	Phototrophy	0.012*	0.022*	0.502
	Plant pathogen	0.055.	0.795	0.145
	Plastic degradation	0.034*	0.679	0.099.
	Respiration of sulfur compounds	0.382	0.008**	0.030*
	Sulfate respiration	0.004**	<0.001***	0.003**
	Sulfur respiration	<0.001***	0.702	0.594
	Ureolysis	0.002**	0.691	0.167
Fungi	Animal parasite-Fungal parasite	0.306	0.306	0.182
	Fungal parasite	0.123	0.291	0.047*
	Fungal parasite-Litter saprotroph	0.149	0.327	0.080.
	Insect parasite-Undefined saprotroph	0.033*	0.033*	0.033*
	Animal pathogen-Endophyte-Epiphyte-Fungal parasite-Plant pathogen-Wood saprotroph	0.381	0.945	0.187
	Dung saprotroph-Soil saprotroph-Wood saprotroph	0.13	0.144	0.134
	Dung saprotroph-Undefined saprotroph-Wood saprotroph	0.175	0.100.	0.148

*n* = 4. represents 0.05 < *p* < 0.1; *represents 0.01 < *p* < 0.05; ** represents 0.001 < *p* < 0.01; *** represents *p* < 0.001.

## 4. Discussion

Changes in precipitation patterns can alter the composition and abundance of belowground microbial communities (Zhou et al., 2017; Engelhardt et al., 2018). Consistent with this, our results showed bacterial community composition was significantly altered by precipitations as demonstrated by NMDS, adonis and ANOSIM results (Figure 2A). Specifically, we observed that the bacterial community in plots with increased precipitation amounts in the late growing season were clearly separated from that in control and in plots with increased precipitation amounts in the early growing season. These results indicated that increasing precipitation in the late growing season might have a more pronounced effect on bacterial community. In addition, alpha-diversity indices of bacterial communities did not significantly change with increased precipitation amounts in the early growing season whereas there were significant decreases with increased precipitation amounts in the late growing season (Figures 1A–D). This finding contrasts with previous studies reporting that bacterial alpha-diversity always increases with increased precipitation amounts in desert ecosystems (Huang et al., 2018; Cheng et al., 2021; Gao et al., 2021). Thus, our study suggested that changes of bacterial diversity are also related to the timing of increased precipitation.

There are two possible reasons for this finding. The first reason is the close association between bacterial community and the vegetation growth (Zhou et al., 2017). A previous study found that if there were adequate water supplementation during the early growing season, plants would grow vigorously (Bunting et al., 2021), which would provide more substrate for bacteria. Although there were higher precipitation amounts and soil water availability in the late growing season, plant growth and root development could not be enhanced as much as in the early growing season treatment plots (Ru et al., 2018). Thus, we observed higher bacterial alpha-diversity in increased precipitation amounts in the early growing season plots than that in the late growing season treatment plots. Consistent with these findings, we observed an increased trend of plant coverage, although it was not statistically significant due to the large coefficient of variation caused by high heterogeneity in desert ecosystems (Supplementary Table S3).

The second possible explanation for the relationship between bacterial diversity and the timing of increased precipitation is linked to the adaptation of the bacterial community to wet conditions over short time periods. Prior research has demonstrated that elevated levels of precipitation can boost soil microbial activity, but these effects tend to diminish as experiments proceed in arid and semi-arid regions (Evans and Wallenstein, 2012; Ma et al., 2012). In our study, following a prolonged period of severe drought during the dry season, the addition of water in the early growing season could potentially activate a range of soil microbes. However, the soil microbes in plots with increased precipitation during the late growing season may have been weakened by previous exposure to natural precipitation in the early growing season. Thus, even though there was greater water addition during the late growing season, it did not activate soil microbes as extensively as those in the treatment plots during the early growing season.

In addition, the observations that the alpha-diversity in the early growing season did not significantly change might attribute to two main reasons. First, the increased precipitation in the early growing season fostered plant growth and facilitated root development, consequently instigating bacterial proliferation. Nevertheless, this increase in precipitation also resulted in heightened water demands from the plants, consequently culminating in a discernible decline in soil moisture levels within the context of the early growing season treatment, precisely aligned with the juncture of soil sample collection at the denouement of the growing season (as delineated in Supplementary Table S2). This reduction of soil moisture could limit the growth of some bacteria, which offset the positive effects seen in the early growing season. In addition, alpha-diversity remained unchanged in the early growing season treatments throughout the entire growing season. Secondly, bacterial community exhibited a high resilience to disturbance (Werner et al., 2011). Soil samples were collected at the end of the entire growing season, and there was the possibility that the bacterial community in the early growing season treatments may regain its community structure as control since our study only conducted for 5 years.

Moreover, the two-way PERMANOVA analysis results also suggested that bacterial communities may be more sensitive to the timing of increasing precipitation than the amounts of precipitation (season: *p* = 0.001; amounts: *p* = 0.006, Table 1). In contrast, the fungal alpha-diversity index did not change significantly under different treatments (Figures 1E–H), indicating that the desert fungal community is relatively stable and less sensitive to climate change than the bacterial community (Table 1). This is consistent with previous studies conducted in the Chihuahuan desert (Clark et al., 2009), which also indicated a strong ability of fungal communities to remain stable in response to environmental change (Barnard et al., 2013). The high tolerance of fungal communities to water changes in desert soils may be attributed to their ability to produce mycelium at low soil water content (Allen and Kitajima, 2013), which enables them to obtain water and nutrients from distant locations under dry micropore conditions.

Our findings revealed that Proteobacteria, Actinobacteria and Firmicutes were the most dominant taxa in the bacterial community, which is consistent with our previous research (Gao et al., 2021). Furthermore, we found that in the early growing season, the relative abundance of abundant taxa remained relatively stable. However, during the late growing season, the relative abundance of copiotrophic bacteria (e.g., Proteobacteria) significantly increased while oligotrophic groups (e.g., Chloroflexi) decreased with increased precipitation amounts (Figure 2C; Table 2). Analogous alterations in the proportional representation of bacterial phyla have been observed in precipitation manipulation experiments carried out within forest and polar ecosystems (Fierer et al., 2003; Buelow et al., 2016). This can be explained by the increased water availability, which might stimulate the mineralization of soil organic matter in increased precipitation amounts in the late growing season treatments (Austin et al., 2004; Grigg et al., 2008), and promote the growth of copiotrophic taxa. Conversely, in the early growing season treatments where the water availability was like the control plots, the taxa remain stable, suggesting that soil moisture has played a role in shaping bacterial community structure. Unlike the changes in the bacterial community, the fungal community at the phylum level did not show any significant change (Figure 2D; Table 2). The dominance of Ascomycota in fungal community is consistent with other studies conducted in different ecosystems (She et al., 2018; Shi et al., 2020; Wang et al., 2021). This may be related to the ability of Ascomycota to survive in harsh ecological environments and even to convert radiation into metabolic activity (Dadachova et al., 2007). Both mantel test and random forest results revealed that soil nitrogen (NO_3_^−^-N and TN) and moisture were the main factors shaping the community composition of bacteria and fungi (Supplementary Figures S2, S3), which is consistent with the response of precipitation amounts to microbial communities in Inner Mongolian temperate grasslands and a desert Shrubland (Wang et al., 2015; She et al., 2018; Wang D. et al., 2018). It is not surprising as soil moisture and nitrogen levels are known to commonly influence the composition and structure of microbial communities in deserts (Julie Laity, 2009; Bachar et al., 2012; Makhalanyane et al., 2015) and other ecosystems (Parker and Schimel, 2011; Zhang et al., 2014; Li X. et al., 2016). Soil moisture and nitrogen were also found to be the first and second limiting factors affecting plant growth in desert. Although we did not directly measure plant biomass since the desert ecosystem is very fragile, other studies have shown that plant biomass does indeed increase during periods of increased water availability (She et al., 2016; Bao et al., 2020). Thus, it can be inferred that microbial community might be indirectly affected by plant growth.

In desert soils, bacterial and fungal members are subjected to low levels of water and high levels of desiccation (Kim et al., 2018). Increased precipitation could force microbes to allocate resources differently and alter microbial community composition, thereby affecting C and N flows and ecosystem functioning significantly (Schimel et al., 2007). Indeed, our study showed that only increased precipitation amounts in the late growing season treatments had significant effects on the functional groups of communities associated with soil nutrient cycling (Figure 4). This indicates that both the increasing of precipitation amount and the season were critical factor affecting potential bacteria community functions (Clark et al., 2009; Huang et al., 2018; Bao et al., 2020). This observation was confirmed by the results of our two-way PERMANOVA analysis of potential microbial community functions. Precipitation increase time had a significant effect (*p* < 0.05) on 14 of the 22 potential functional groups, while precipitation increase amount had a significant effect (*p* < 0.05) on only five of these potential functional groups (Table 4). Some potential functions related to soil nutrient cycling, including aerobic chemoheterotrophy, methylotrophy, cellulolysis, and ureolysis, were significantly affected the timing of precipitation time (0.001 < *p* < 0.05). This may be due to the fact that temperature is significantly affected by the timing of the increase in precipitation (*p* < 0.001) (Supplementary Table S4), whereas previous studies have shown that these organic matter synthesis and decomposition-related reactions are significantly affected by temperature (Liu et al., 2017; Zhou et al., 2017; Xu et al., 2021). In the fungal community, function prediction further demonstrated significant differences between the early growing season and the late growing season in terms of guild (Figure 4). This shows the cumulative effect of different precipitation time on specific fungal communities over 5 years. Previous studies have shown that saprophytic fungi are the primary decomposers of dead plants and roots in the soil (Voriskova and Baldrian, 2013), which play important roles in promoting organic matter and nutrient cycling (Nilsson et al., 2019). Therefore, the increase of the proportions of the saprotrophic fungi might improve the formation of soil organic matter and accelerate nutrition cycling in the early growing season treatments. Although functional predictions could provide us with microscopic views of the microbiome function, we only obtained information on the preliminary steps of metabolite biosynthesis due to the limitations of function prediction technology. In future studies, a deeper investigation involving functional genes and function dynamics (such as biosynthesis of metabolites) should be conducted through shotgun sequencing.

## 5. Conclusion

In this study, we observed varying effects of the different timing of increased precipitation on the bacterial and fungal community structure of desert soils during the early and late growing season. The abundance and diversity of bacterial communities decreased significantly in response to precipitation increase treatment in the late growing season, whereas there were no significant effects on soil fungal community. The difference may be attributed to characteristics of plant development periods during the early and late growing season. Our findings suggest that soil moisture, soil NO_3_^−^-N content and soil total nitrogen content are the primary environmental factors influencing the structure of bacterial and fungal communities in desert soils. Two-way PERMANOVA analysis revealed that precipitation increase time had a greater impact on bacterial community alpha diversity and bacterial community composition compared to precipitation increase amount. Therefore, experiment settings involving a uniform increase or decrease in precipitation throughout the growing season may not accurately reflect the overall response patterns in these systems. Seasonal variation will play a critical role in the response of these communities, and precipitation increase time should be considered when we design future microbial community assessments in desert systems.

## Data availability statement

The datasets presented in this study can be found in online repositories. The names of the repository/repositories and accession number(s) can be found in the article/Supplementary material.

## Author contributions

YG and BW: conceptualization and supervision. YG, XX, and FB: methodology. YX and KY: data analysis. YX: writing—original draft preparation and visualization. YG, XX, FB, JZ, and KY: writing—review and editing. BW: project administration. YG, BW, and JZ: funding acquisition. All authors have read and agreed to the published version of the manuscript.

## Funding

This work was financially supported by the National Natural Science Foundation of China (Grant nos. 32201413, 31400421, and 31600394), the Surplus Funds of Institute of Desertification Studies, CAF (Grant nos. IDS2018JY-3 and IDS2018JY-9), and Scientific Research Fund of Yunnan Provincial Department of Education (No. 2022J0512), Yunnan Province first-class disciplines forestry science discipline construction funds (523003), and Provision for the construction of first-class specialties in forestry science in Yunnan Province (523002).

## Conflict of interest

The authors declare that the research was conducted in the absence of any commercial or financial relationships that could be construed as a potential conflict of interest.

## Publisher’s note

All claims expressed in this article are solely those of the authors and do not necessarily represent those of their affiliated organizations, or those of the publisher, the editors and the reviewers. Any product that may be evaluated in this article, or claim that may be made by its manufacturer, is not guaranteed or endorsed by the publisher.

## Footnotes

^1^ http://ccb.jhu.edu/software/FLASH/

^2^ http://qiime.org/index.html

^3^ http://drive5.com/uchime/uchime_download.html

^4^ http://www.drive5.com/usearch/manual/uchime_algo.html

^5^ http://drive5.com/uparse

^6^ www.R-project.org

^7^ https://Github.com/cran/randomForests

^8^ https://CRAN.R-project.org/package=pheatmap
