# Supplementary material for: The influence of precipitation timing and amount on soil microbial community in a temperate desert ecosystem
Source: Front Microbiol. 2023 Sep 7;14:1249036. doi: 10.3389/fmicb.2023.1249036 (PMC10512721; doi:10.3389/fmicb.2023.1249036)
Supplement: Supplementary file 1 [file Data_Sheet_1.pdf]

Supplementary material

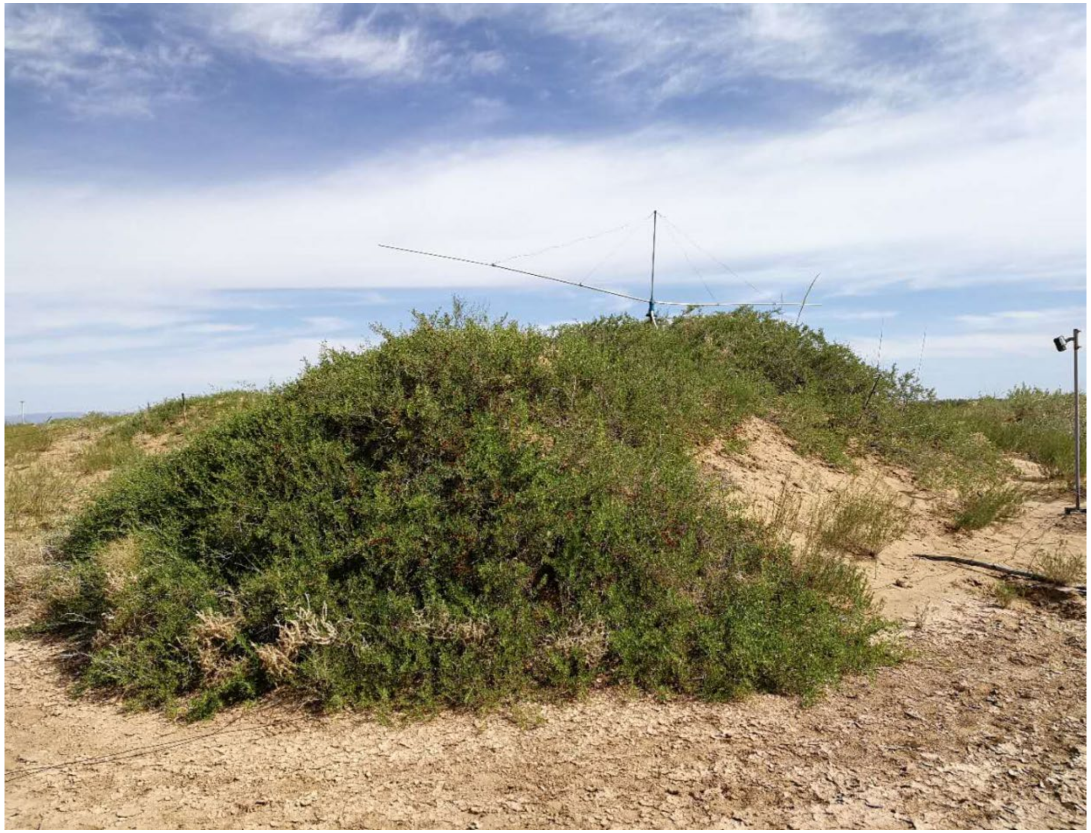

Figure S1 Precipitation increase treatments were conducted using an irrigation system.

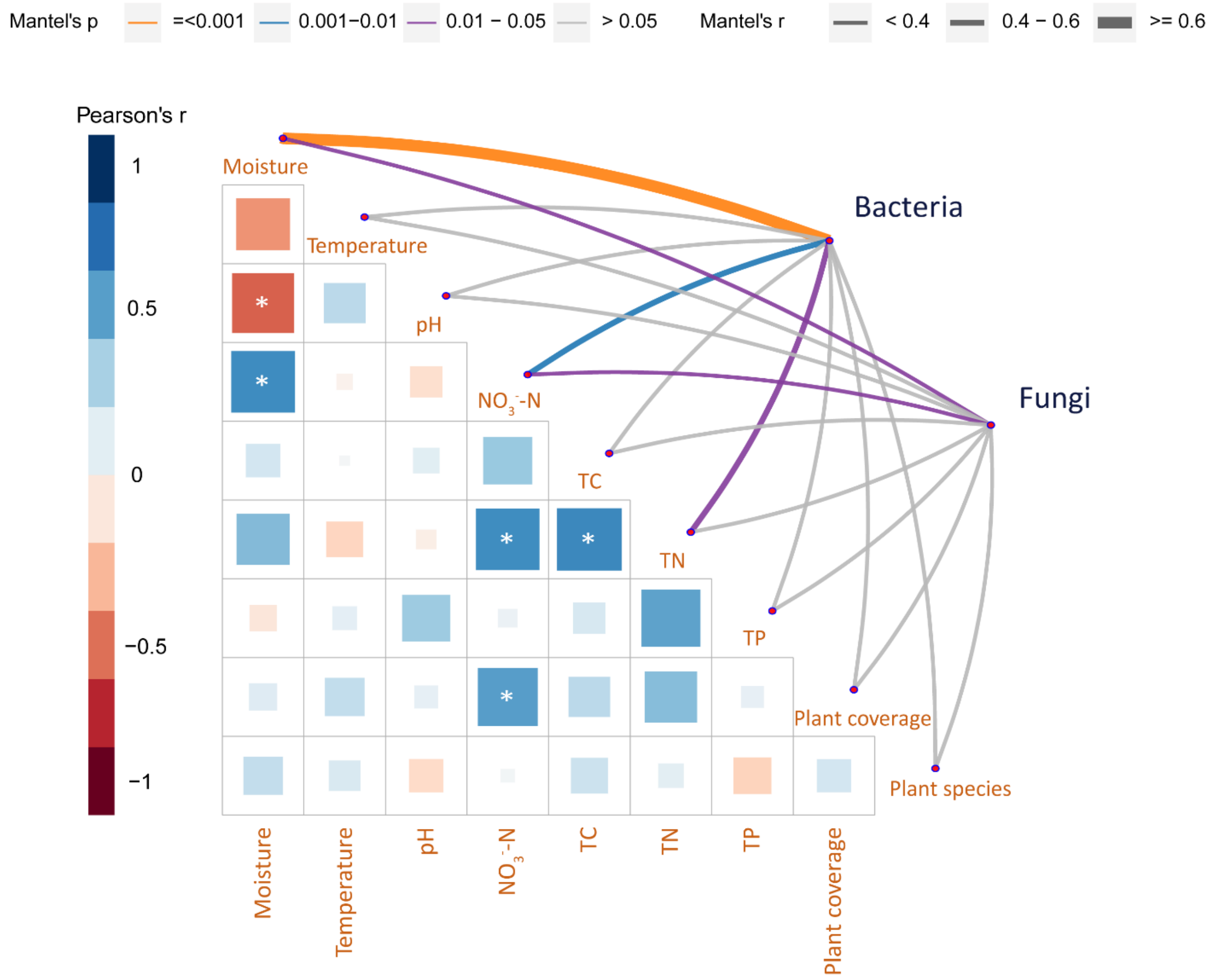

Figure S2 Mantel's correlation test between bacterial and fungal communities and environmental factors.

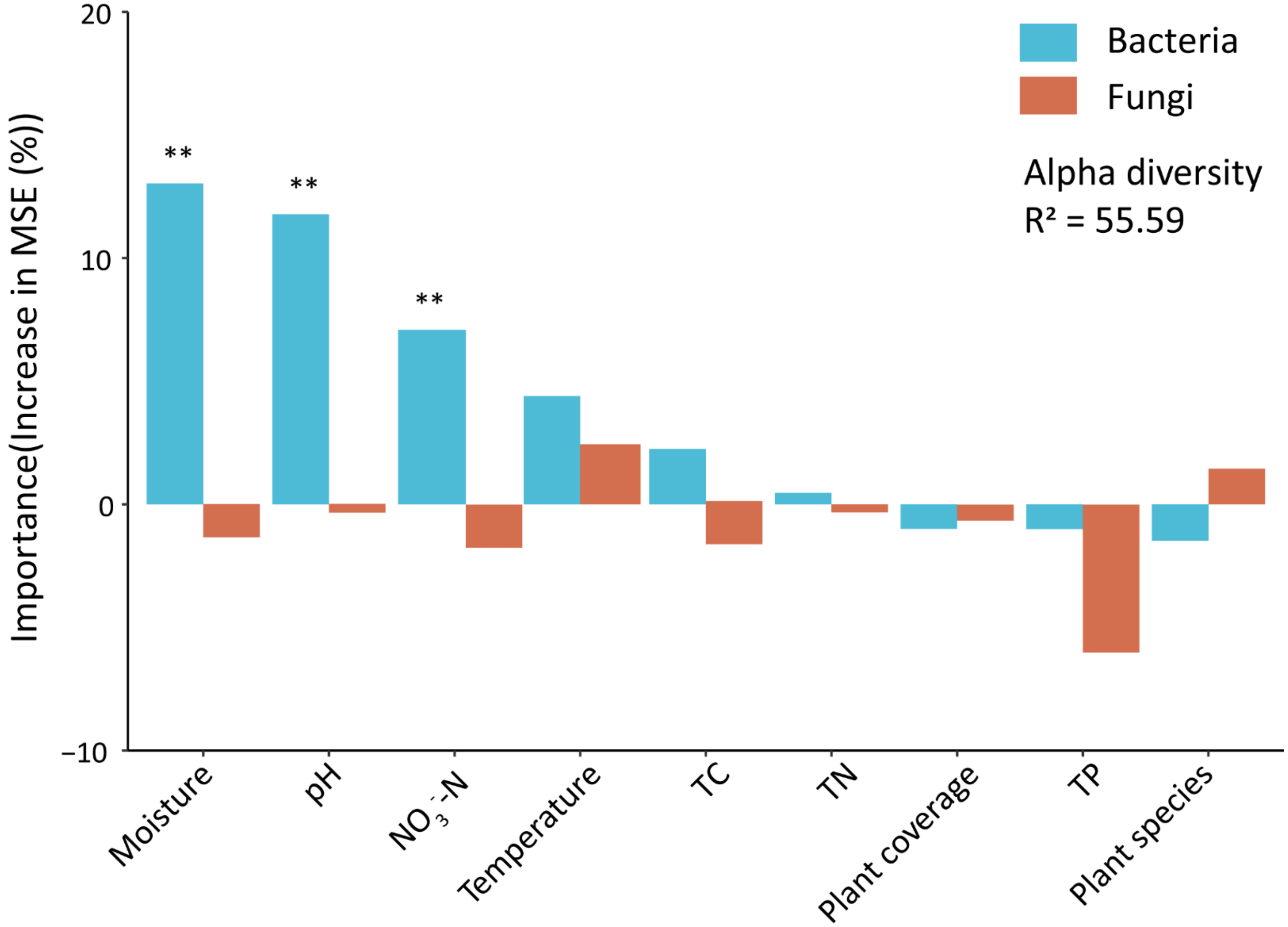

Figure S3 The random forest model demonstrates the effects of environmental factors on the alpha diversity of bacteria and fungi.

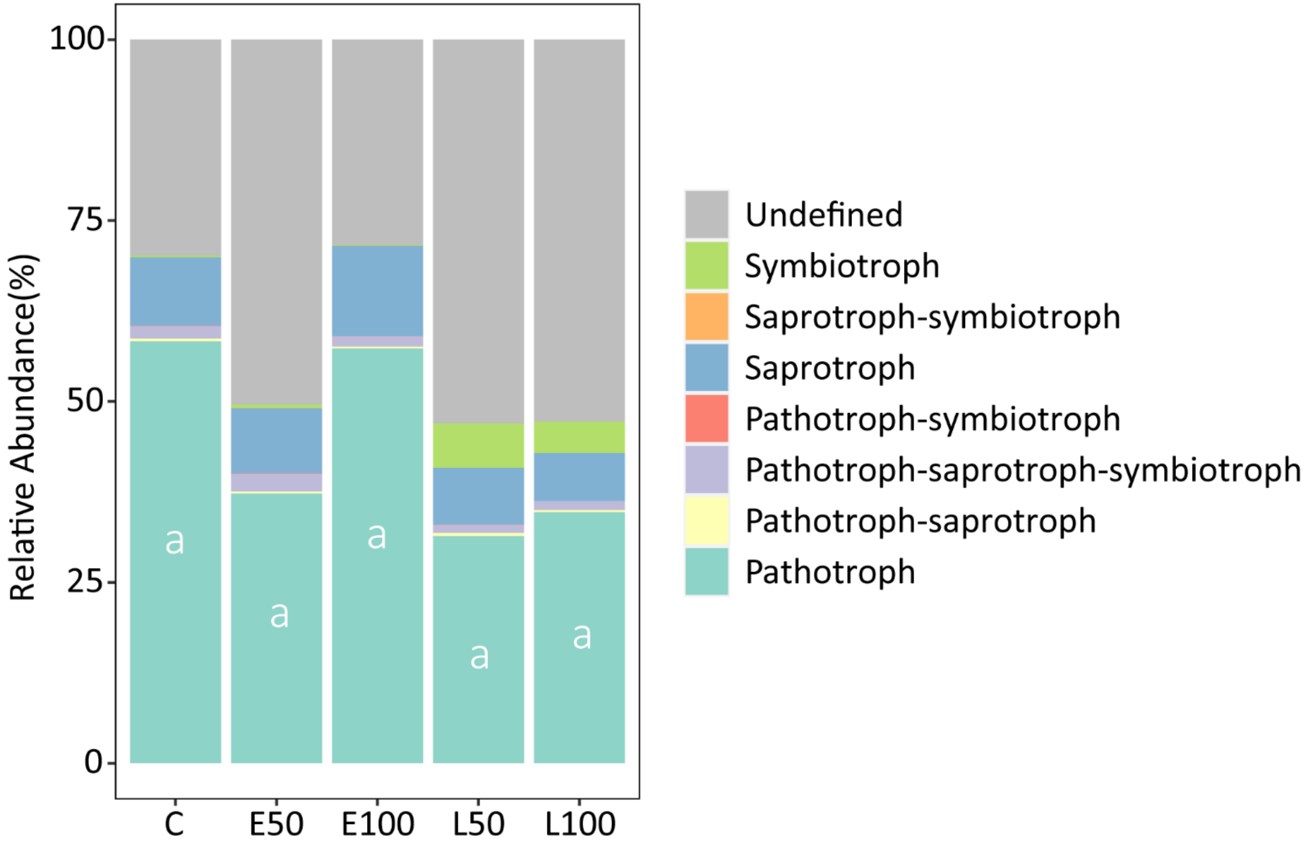

Figure S4 Function prediction of fungal community at trophic Mode level.

Table S1 Experiment design

	Precipitation period					Total water added (mm)	Water amount per time (mm)
	May	June	July	August	September		
C							
E50	25th	10th and 25th	10th			72.500	18.125
E100	25th	10th and 25th	10th			145.000	36.250
L50			25th	10th and 25th	10th	72.500	18.125
L100			25th	10th and 25th	10th	145.000	36.250

Control (C) = ambient precipitation; E50 = ambient precipitation +50% of the local annual average precipitation in the early growing reason, E100 = ambient precipitation +100% of the local annual average precipitation in the early growing reason, L50 = ambient precipitation +50% of the local annual average precipitation in the late growing reason, L100 = ambient precipitation +100% of the local annual average precipitation in the late growing reason.

Table S2 Effects of precipitation increase on soil and plant properties. Values represent mean values and standard deviations (n = 4).

	C	E50	E100	L50	L100
Moisture (0-20 cm, %)	3.060	2.290	2.590	4.270	7.170
Temperature (0-20 cm, °C)	20.992	23.052	24.980	20.117	21.564
pH	**8.033±0.068a**[1]	**8.033±0.069a**	**7.945±0.025ab**	**7.855±0.037b**	**7.883±0.01b**
NO_3^- ($mg{\cdot}g^{-1}$)	**0.276±0.078b**	**0.255±0.074b**	**0.306±0.060b**	**0.282±0.077b**	**0.438±0.100a**
TC ($mg{\cdot}g^{-1}$)	0.167±0.061a	0.174±0.024a	0.155±0.041a	0.143±0.036a	0.190±0.049a
TN ($mg{\cdot}g^{-1}$)	0.019±0.005a	0.016±0.002a	0.017±0.002a	0.018±0.001a	0.021±0.006a
TP ($mg{\cdot}g^{-1}$)	0.157±0.010a	0.138±0.009a	0.151±0.020a	0.138±0.012a	0.143±0.018a
Plant coverage (%)	40.000±15.811a	62.500±20.207a	51.25±20.565a	41.250±6.292a	61.250±19.311a
Plant species (%)	5.500±1.291a	7.250±0.957a	6.500±1.291a	6.250±1.258a	7.500±1.291a

[1]Values in bold indicate significant responses to precipitation, as assessed by one-way ANOVA. Lowercase letters in bold depict significant differences across treatments.[2]Abbreviations: TC, total carbon; TN, total nitrogen; TP, total phosphorus; Control (C) =ambient precipitation, E50 = ambient precipitation +50% of the local annual average precipitation in the early growing reason, E100 = ambient precipitation +100% of the local annual average precipitation in the early growing reason, L50 = ambient precipitation +50% of the local annual average precipitation in the late growing reason, L100 = ambient precipitation +100% of the local annual average precipitation in the late growing reason.

Table S3. Large variations of the soil properties.

Variable	C		E50		E100		L50		L100	
	Mean	CV (%)	Mean	CV (%)	Mean	CV (%)	Mean	CV (%)	Mean	CV (%)
Moisture	0.031	0	0.023	0	0.026	0	0.043	0	0.072	0
Temperature	20.992	0	23.052	0	24.98	0	20.117	0	21.564	0
pH	8.033	0.853	8.033	0.865	7.945	0.317	7.855	0.471	7.883	0.121
NO_3^-	0.276	28.228	0.255	28.939	0.306	19.56	0.282	27.355	0.438	22.94
TC	0.167	36.37	0.174	14.004	0.155	26.483	0.143	25.44	0.19	25.746
TN	0.019	26.622	0.016	11.649	0.017	12.707	0.018	7.089	0.021	26.166
TP	0.157	6.405	0.138	6.276	0.151	13.287	0.138	8.873	0.143	12.51
Plant coverage	40	39.528	62.5	32.332	51.25	40.127	41.25	15.252	61.25	31.528
Plant species	5.5	23.473	7.25	13.206	6.5	19.861	6.25	20.133	7.5	17.213

Abbreviations: CV, coefficient of variation; TC, total carbon; TN, total nitrogen; TP, total phosphorus; Control (C) =ambient precipitation, E50 = ambient precipitation +50% of the local annual average precipitation in the early growing reason, E100 = ambient precipitation +100% of the local annual average precipitation in the early growing reason, L50 = ambient precipitation +50% of the local annual average precipitation in the late growing reason, L100 = ambient precipitation +100% of the local annual average precipitation in the late growing reason.

Table S4. Results of Two-way ANOVA on the effects of season (S), amount (A) and their interactions on environment factors. (n = 4)

	S	A	S×A
	Pr(>F)	Pr(>F)	Pr(>F)
Moisture	<0.001***	<0.001***	<0.001***
Temperature	<0.001***	<0.001***	<0.001***
pH	<0.001***	0.175	0.017*
NO_3^-	0.069	0.022*	0.211
TC	0.921	0.488	0.114
TN	0.097	0.208	0.406
TP	0.612	0.272	0.612
Plant coverage	0.536	0.629	0.102
Plant species	1.000	0.686	0.124

Abbreviations: S, Precipitation seasonality; A, Amount of precipitation; S×A, Interaction between precipitation seasonality and precipitation amount; TC, total carbon; TN, total nitrogen; TP, total phosphorus; . represents $0.05 < p < 0.1$; * represents $0.01 < p < 0.05$; ** represents $0.001 < p < 0.01$; *** represents $p < 0.001$.

Table S5. Potential functional taxa with significant changes in the bacterial community annotated in the KEGG database.

Level 3	Gene	C	E50	E100	L50	L100
Glycolysis / Gluconeogenesis	ACSS1_2	0.12±0.00a	0.12±0.00a	0.12±0.00a	0.12±0.01a	0.11±0.01b
Galactose metabolism	galE	0.16±0.00ab	0.16±0.01a	0.16±0.01ab	0.15±0.00b	0.14±0.01c
Inositol phosphate metabolism	E3.1.3.25	0.11±0.00a	0.10±0.01a	0.11±0.01a	0.10±0.01ab	0.09±0.00b
Glyoxylate and dicarboxylate metabolism	glnA	0.15±0.00bc	0.16±0.00a	0.15±0.00c	0.16±0.00a	0.16±0.00ab
Butanoate metabolism	E2.2.1.6L	0.16±0.00a	0.16±0.00a	0.16±0.00a	0.16±0.01a	0.14±0.01b
Fatty acid biosynthesis	fabG	0.35±0.00a	0.36±0.01a	0.35±0.01a	0.33±0.01b	0.31±0.02c
	ACSL	0.19±0.00a	0.20±0.01a	0.19±0.01a	0.18±0.01a	0.15±0.03b
	ACADM	0.12±0.01a	0.13±0.01a	0.12±0.01a	0.11±0.01ab	0.10±0.02b
Glycerolipid metabolism	plsC	0.13±0.00a	0.13±0.00a	0.12±0.00b	0.13±0.00a	0.13±0.00a
Glycerophospholipid metabolism	E3.1.4.46	0.13±0.00a	0.13±0.01a	0.13±0.01a	0.12±0.01ab	0.11±0.02b
Purine metabolism	PFAS	0.16±0.00ab	0.16±0.00a	0.16±0.00ab	0.16±0.00b	0.15±0.01c

Cysteine and methionine metabolism	E2.6.1.42	0.11±0.00a	0.10±0.00a	0.10±0.00a	0.10±0.00b	0.09±0.00c
Arginine and proline metabolism	E3.5.1.4	0.13±0.00a	0.14±0.01a	0.14±0.01a	0.12±0.01a	0.10±0.02b
Glutathione metabolism	GST	0.16±0.00bc	0.14±0.01c	0.15±0.02bc	0.17±0.03ab	0.19±0.02a
Peptidoglycan biosynthesis	bcrC	0.11±0.00ab	0.11±0.01a	0.12±0.01a	0.10±0.00b	0.09±0.00c
Aminoacyl-tRNA biosynthesis	gatA	0.12±0.00a	0.12±0.00a	0.12±0.01a	0.12±0.00a	0.11±0.00b
Nucleotide excision repair	uvrD	0.13±0.00ab	0.13±0.00a	0.13±0.00ab	0.12±0.01bc	0.11±0.01c
Two-component system	mcp	0.17±0.00bc	0.17±0.00c	0.18±0.01ab	0.17±0.01bc	0.19±0.02a
Flagellar assembly	rpoD	0.11±0.00a	0.11±0.01a	0.11±0.01a	0.10±0.00b	0.08±0.00c
Quorum sensing	ddpD	0.12±0.00ab	0.12±0.00ab	0.13±0.00a	0.12±0.00b	0.12±0.01b
Peptidases and inhibitors	map	0.10±0.00a	0.11±0.00a	0.11±0.00a	0.10±0.00ab	0.10±0.00b
Transcription factors	parB	0.11±0.00b	0.11±0.00b	0.11±0.00b	0.11±0.01b	0.12±0.01a
	fur	0.11±0.00ab	0.12±0.01a	0.12±0.00ab	0.11±0.00b	0.10±0.01c
	arsR	0.11±0.00a	0.11±0.00a	0.11±0.00a	0.10±0.01a	0.09±0.01b

	fusA	0.11±0.00a	0.11±0.00a	0.11±0.00a	0.10±0.00b	0.10±0.00c
Transcription machinery	rpoE	0.50±0.01ab	0.50±0.01a	0.49±0.01ab	0.48±0.01b	0.47±0.02c
Chromosome and associated proteins	ftsK	0.11±0.00a	0.11±0.01a	0.11±0.00a	0.10±0.01a	0.10±0.01b
	parA	0.16±0.00b	0.16±0.00b	0.15±0.00b	0.16±0.01ab	0.17±0.01a
Transporters	ABC-2.A	0.43±0.00ab	0.44±0.02a	0.43±0.02a	0.40±0.02b	0.36±0.03c
	ABC-2.P	0.39±0.00ab	0.40±0.02a	0.40±0.01a	0.37±0.01b	0.34±0.02c
	ABC.CD.A	0.21±0.00a	0.22±0.01a	0.21±0.01a	0.20±0.01b	0.18±0.02b
	ABC.CD.P	0.21±0.00a	0.22±0.01a	0.21±0.01a	0.20±0.01b	0.18±0.02b
	TC.FEV.OM	0.13±0.00b	0.12±0.01b	0.12±0.01b	0.14±0.03ab	0.18±0.05a
	ABC.PA.P	0.15±0.01bc	0.14±0.01c	0.14±0.01bc	0.16±0.01ab	0.17±0.01a
	ABC.PA.S	0.16±0.01b	0.15±0.01b	0.15±0.01b	0.17±0.01ab	0.17±0.01a
